# Pre-adolescence DNA methylation is associated with lung function trajectories from pre-adolescence to adulthood

**DOI:** 10.1186/s13148-020-00992-5

**Published:** 2021-01-06

**Authors:** Shadia Khan Sunny, Hongmei Zhang, Fawaz Mzayek, Caroline L. Relton, Susan Ring, A. John Henderson, Susan Ewart, John W. Holloway, S. Hasan Arshad

**Affiliations:** 1 Division of Epidemiology, Biostatistics, and Environmental Health Sciences, School of Public Health, University of Memphis, Memphis, TN 38152 USA; 2 MRC Integrative Epidemiology Unit, University of Bristol, Bristol, BS8 2BN UK; 3 Population Health Sciences, University of Bristol, Bristol, BS8 2BN UK; 4 Large Animal Clinical Sciences, Michigan State University, East Lansing, MI USA; 5 Human Development and Health, Faculty of Medicine, University of Southampton, Southampton, SO16 6YD UK; 6 NIHR Southampton Biomedical Research Centre, University Hospital Southampton, Southampton, SO16 6YD UK; 7 Clinical and Experimental Sciences, Faculty of Medicine, University of Southampton, Southampton, SO16 6YD UK; 8 The David Hide Asthma and Allergy Research Centre, St Mary’s Hospital, Parkhurst Road, Newport, Isle of Wight, PO30 5TG UK

**Keywords:** Epigenome-wide, Adolescence, Lung function trajectory, DNA methylation, COPD, Population-based cohorts (IOW birth cohort, ALSPAC)

## Abstract

**Background:**

The pattern of lung function development from pre-adolescence to adulthood plays a significant role in the pathogenesis of respiratory diseases. Inconsistent findings in genetic studies on lung function trajectories, the importance of DNA methylation (DNA-M), and the critical role of adolescence in lung function development motivated the present study of pre-adolescent DNA-M with lung function trajectories. This study investigated epigenome-wide associations of DNA-M at cytosine-phosphate-guanine dinucleotide sites (CpGs) at childhood with lung function trajectories from childhood to young adulthood.

**Methods:**

DNA-M was measured in peripheral blood at age 10 years in the Isle of Wight (IOW) birth cohort. Spirometry was conducted at ages 10, 18, and 26 years. A training/testing-based method was used to screen CpGs. Multivariable logistic regressions were applied to assess the association of DNA-M with lung function trajectories from pre-adolescence to adulthood. To detect differentially methylated regions (DMRs) among CpGs, DMR enrichment analysis was conducted. Findings were further tested in the Avon Longitudinal Study of Parents and Children (ALSPAC) cohort. Pathway analyses were performed on the mapped genes of the identified CpGs and DMRs. Biological relevance of the identified CpGs was assessed with gene expression. All analyses were stratified by sex.

**Results:**

High and low trajectories of FVC, FEV_1_, and FEV_1_/FVC in each sex were identified. At *P*_*Bonferroni*_ < 0.05, DNA-M at 96 distinct CpGs (41 in males) showed associations with FVC, FEV_1_, and FEV_1_/FVC trajectories in IOW cohort. These 95 CpGs (cg24000797 was disqualified) were further tested in ALSPAC; 44 CpGs (19 in males) of these 95 showed the same directions of association as in the IOW cohort; and three CpGs (two in males) were replicated. DNA-M at two and four CpGs showed significant associations with the corresponding gene expression in males and females, respectively. At *P*_*FDR*_ < 0.05, 23 and 10 DMRs were identified in males and females, respectively. Pathways were identified; some of those were linked to lung function and chronic obstructive lung diseases.

**Conclusion:**

The identified CpGs at pre-adolescence have the potential to serve as candidate markers for lung function trajectory prediction and chronic lung diseases.

## Introduction

The patterns of lung function development, from pre-adolescence to adulthood, play a major role in the pathogenesis of respiratory diseases. Recent studies have highlighted that reduced lung function development in young adulthood predisposes to respiratory and other chronic diseases in later life and is also associated with early mortality [1, 2]. Lung function grows dramatically throughout childhood and reaches its peak in adolescence or early adulthood. After a brief period of stable lung function in early adulthood, a gradual decline ensues with aging [3–5]. Previous studies have demonstrated that early decline of lung function, and/or failure to reach maximal level lung function (even with a normal rate of decline), is associated with the development of chronic obstructive pulmonary disease (COPD) in later life [3–6], suggesting that the origins of COPD lie, in part, in early life [6, 7]. COPD is projected to become the third leading cause of death worldwide by 2030 [8, 9], highlighting how insights into the trajectories of lung function development from childhood-to-young adulthood would be beneficial for COPD prediction, prevention, and management.

Encouraged by the significance and advantage of longitudinal designs, we and others examined the temporal trend of lung function growth and decline through multiple important stages of life: childhood, adolescence, and adulthood [4, 10–12]. These studies demonstrated that there are distinct groups of individuals with a persistently low lung function trajectory from childhood-to-adulthood, suggested a potential connection with COPD in later life. One study showed weak evidence that persistently low FEV_1_ trajectory is associated with genetic factors in addition to multiple childhood exposures [10]. A recent study based on repeated measurement of lung function in adults reported that genetic variants associated with cross-sectional lung function measurements were not associated with a longitudinal decline of lung function [13]. These inconsistent findings in genetic studies and the clear impact of environmental factors on lung function motivated the investigation of the role of epigenetic factors such as DNA methylation (DNA-M) in determining variation in lung function between people and over time.

DNA-M represents an epigenetic mechanism that regulates gene expression, which consequently influences disease risk [14, 15]. Growing evidence indicates that DNA-M in whole blood is associated with lung function and its related diseases such as asthma and COPD [15–18]. Pre-adolescence adverse exposure is shown to be associated with adulthood chronic lung diseases [19]. As an epigenetic memory of past exposures, the role of pre-adolescence DNA-M on lung function trajectories from pre-adolescence to young adulthood is unknown [19]. We hypothesized that differential methylation at certain cytosine-phosphate-guanine dinucleotide sites (CpGs) in childhood is associated with the trajectories of lung function. Given that lung function growth and decline is sex-dependent and such dependence is attributable to multiple biological determinants, including dimensional/anatomical, immunological, and hormonal determinants [20–23], we examined the hypothesis in male and female participants, separately [12, 24]. The study was carried out in the birth cohort located on the Isle of Wight (IOW) in the UK. To assess the potential of generalizability, an independent UK birth cohort, the Avon Longitudinal Study of Parents and Children (ALSPAC) cohort, was used for replication.

## Results

**Fig. 1:**
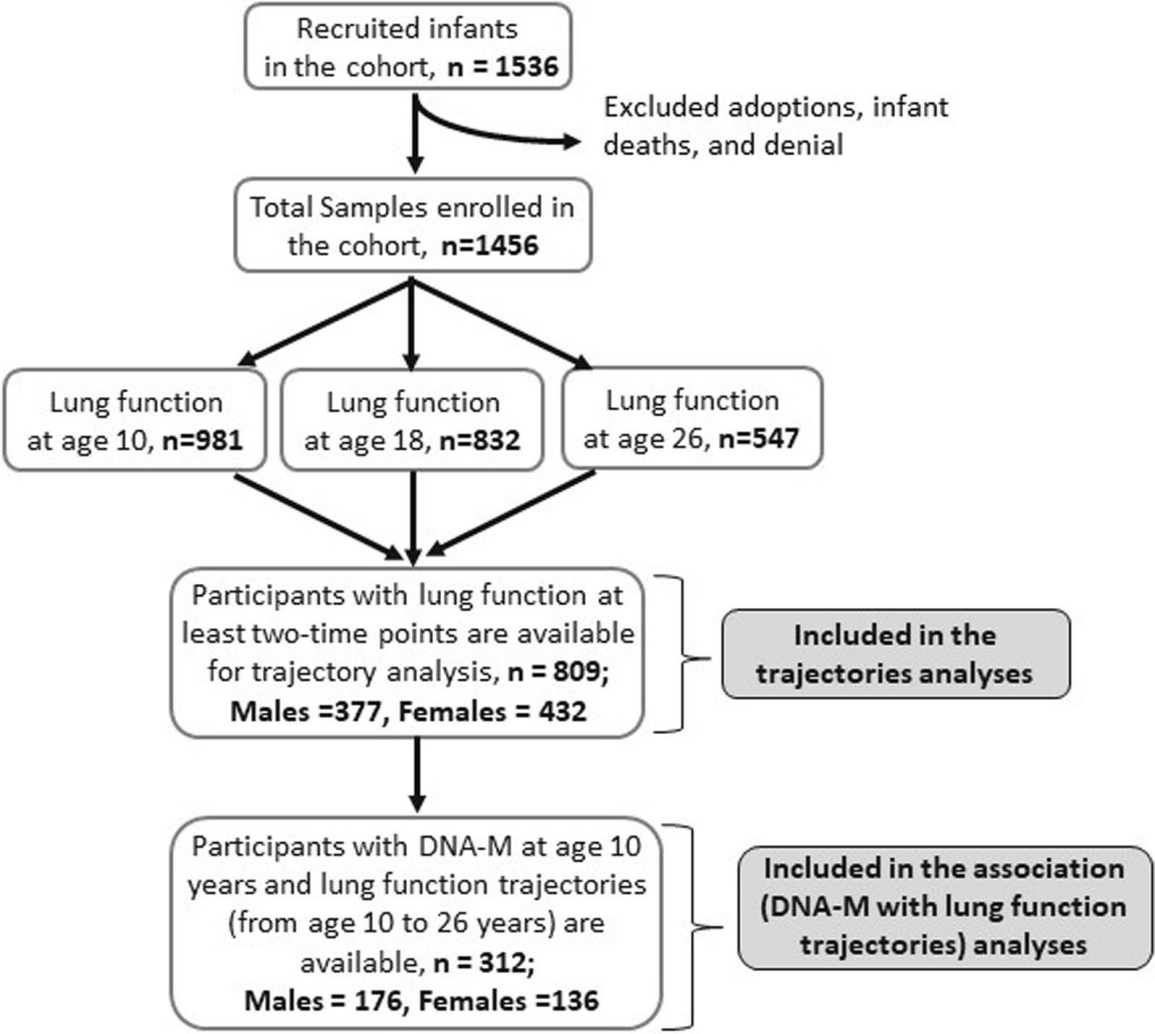
Flow chart of study participants of the IOW cohort for each step of the analysis

In the complete IOW cohort (*n* = 1456), lung function measurements at ages 10, 18, and 26 years were available for 980 (67.3%), 838 (57.6%), and 547 (37.6%) participants, respectively. A total of 377 male and 432 female participants were included for trajectory analyses, and each of the participants had spirometry tests at two or more of the three ages (Fig. 1). The analysed sub-sample (*n* = 809) was not statistically different from the complete cohort (*n* = 1456) regarding FVC, FEV_1_, and FEV_1_/FVC at the corresponding ages (Table 1).

**Table 1:** Comparison of lung function measurements of enrolled participants and participants included in the analyses

Lung function	Males			Females		
	Enrolled sample (*N* = 786) Mean ± SD	Analytical sample (*N* = 377) Mean ± SD	*P* value	Enrolled sample (*N* = 750) Mean ± SD	Analytical sample (*N* = 432) Mean ± SD	*P* value
Age 10						
FVC (L)	2.35 ± 0.34	2.37 ± 0.34	0.385	2.24 ± 0.33	2.24 ± 0.33	0.862
FEV_1_ (L)	2.06 ± 0.30	2.07 ± 0.30	0.405	2.00 ± 0.29	2.00 ± 0.30	0.991
FEV_1_/FVC	0.87 ± 0.06	0.88 ± 0.06	0.831	0.90 ± 0.05	0.89 ± 0.06	0.620
Age 18						
FVC (L)	5.35 ± 0.72	5.36 ± 0.72	0.853	3.96 ± 0.53	3.97 ± 0.52	0.591
FEV_1_ (L)	4.61 ± 0.62	4.61 ± 0.63	0.916	3.47 ± 0.45	3.48 ± 0.45	0.745
FEV_1_/FVC	0.87 ± 0.07	0.86 ± 0.07	0.597	0.88 ± 0.07	0.88 ± 0.07	0.679
Age 26						
FVC (L)	5.85 ± 0.82	5.88 ± 0.83	0.588	4.24 ± 0.54	4.25 ± 0.54	0.694
FEV_1_ (L)	4.60 ± 0.72	4.64 ± 0.73	0.582	3.42 ± 0.43	3.43 ± 0.42	0.894
FEV_1_/FVC	0.79 ± 0.72	0.79 ± 0.07	0.904	0.81 ± 0.06	0.81 ± 0.06	0.662

### Lung function trajectories

**Fig. 2:**
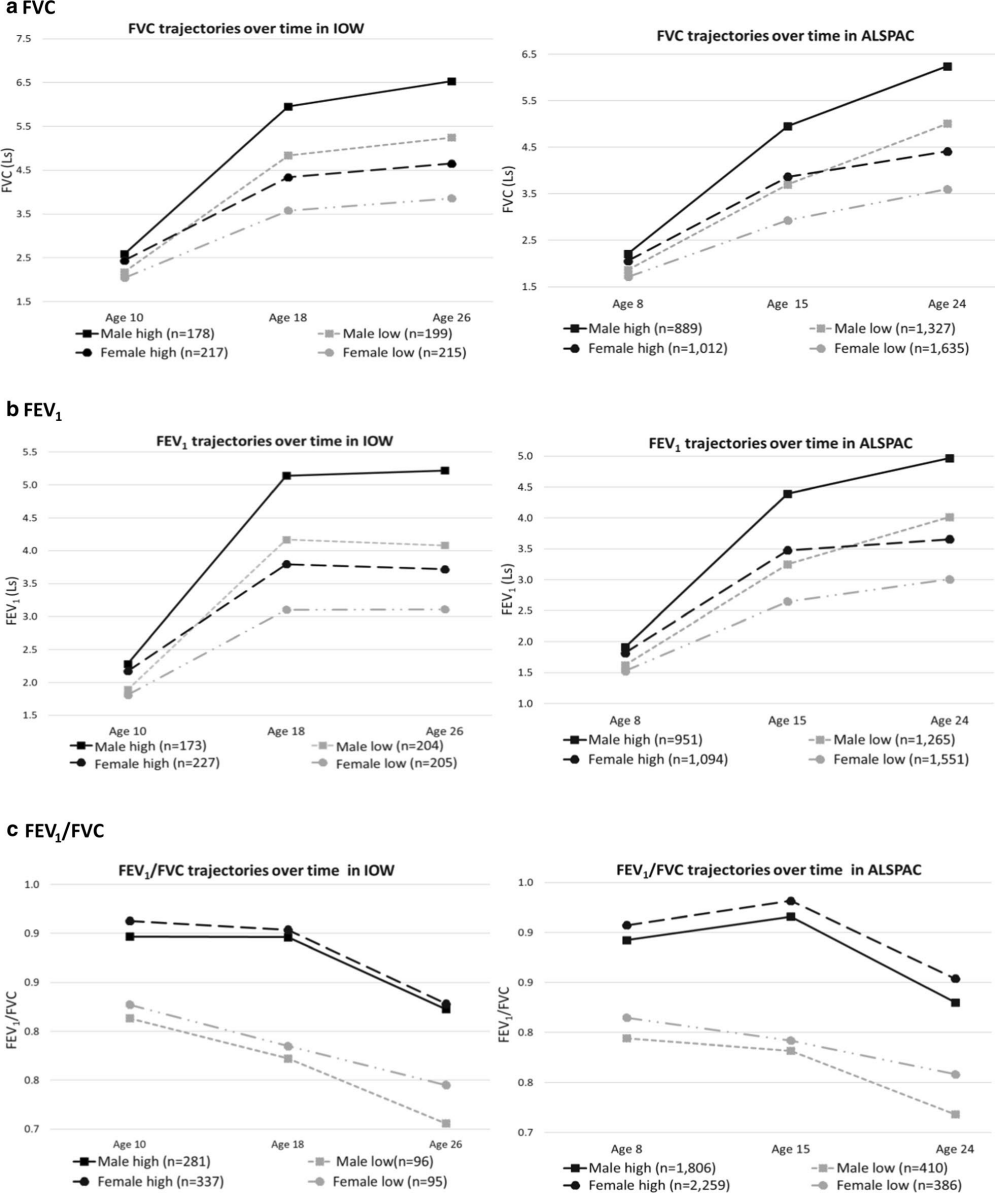
Distinct lung function trajectories from childhood-to-adulthood following comparable patterns in the IOW and ALSPAC cohorts. *Note*: Among the subjects assigned to each trajectory with a probability > 0.5, most assignments were with a probability ≥ 0.7, much higher than 0.5. In the following, we provide, for each sex and lung function parameter, the percentages of assignments with an assignment probability ≥ 0.7: (1) Among the male participants who were assigned to persistently low lung function trajectories with a probability > 0.5, 173 of 199 (86.9%) for FVC; 178 of 204 (87.3%) for FEV_1_, and 79 of 96 (82.3%) for FEV_1_/FVC had a probability ≥ 0.7 of belonging to their trajectory class. (2)The males assigned to high lung function trajectories with a probability > 0.5, 165 of 178, (92.7%) for FVC, 156 of 173 (90.2%) for FEV_1_, and 267 of 281 (95.0%) for FEV_1_/FVC had a probability ≥ 0.7 of belonging to the high lung function trajectory group. (3) Among the female participants assigned to each trajectory with a probability > 0.5, 190 of 215 (88.4%) for FVC, 188 of 205 (91.7%) for FEV_1_, and 78 of 95 (82.1%) for FEV_1_/FVC in persistently low lung function trajectory group had a probability ≥ 0.7 of belonging to their trajectory class. (4) The females assigned to persistently high lung function trajectories with a probability > 0.5, 195 of 217, (89.9%) for FVC, 191 of 227 (84.1%) for FEV_1_, and 311 of 337 (92.3%) for FEV_1_/FVC had a probability ≥ 0.7 of belonging to the normal lung function trajectory group

In trajectory analyses, two distinct lung function trajectories from pre-adolescence to adulthood (10 to 26 years of age) were identified in the IOW cohort, labelled as ‘low trajectory’ and ‘high trajectory’ for FVC, FEV_1_, and FEV_1_/FVC in both male and female participants (Fig. 2). Among the 377 male participants, 199 (52.8%), 204 (54.1%), and 96 (25.5%) were assigned to low FVC, FEV_1_, and FEV_1_/FVC trajectories (Fig. 2: gray “dashed lines”), respectively, using probability > 0.5 to define class membership. Among these male participants, at least 82% and 92% of them had a trajectory assignment probability ≥ 0.7 for low and high trajectories, respectively.

Similarly, among the 432 female participants, 215 (49.8%), 205 (47.5%), and 95 (22.0%) were assigned to low FVC, FEV_1_, and FEV_1_/FVC trajectories, respectively (Fig. 2: gray “two-dot-dashed lines”) using probability > 0.5 to define the group. More than 82% and 84% of the female participants had a probability ≥ 0.7 of being assigned to the low and high trajectories, respectively, for all three lung function parameters.

### Pre-adolescence DNA-M and lung function trajectories

**Fig. 3:**
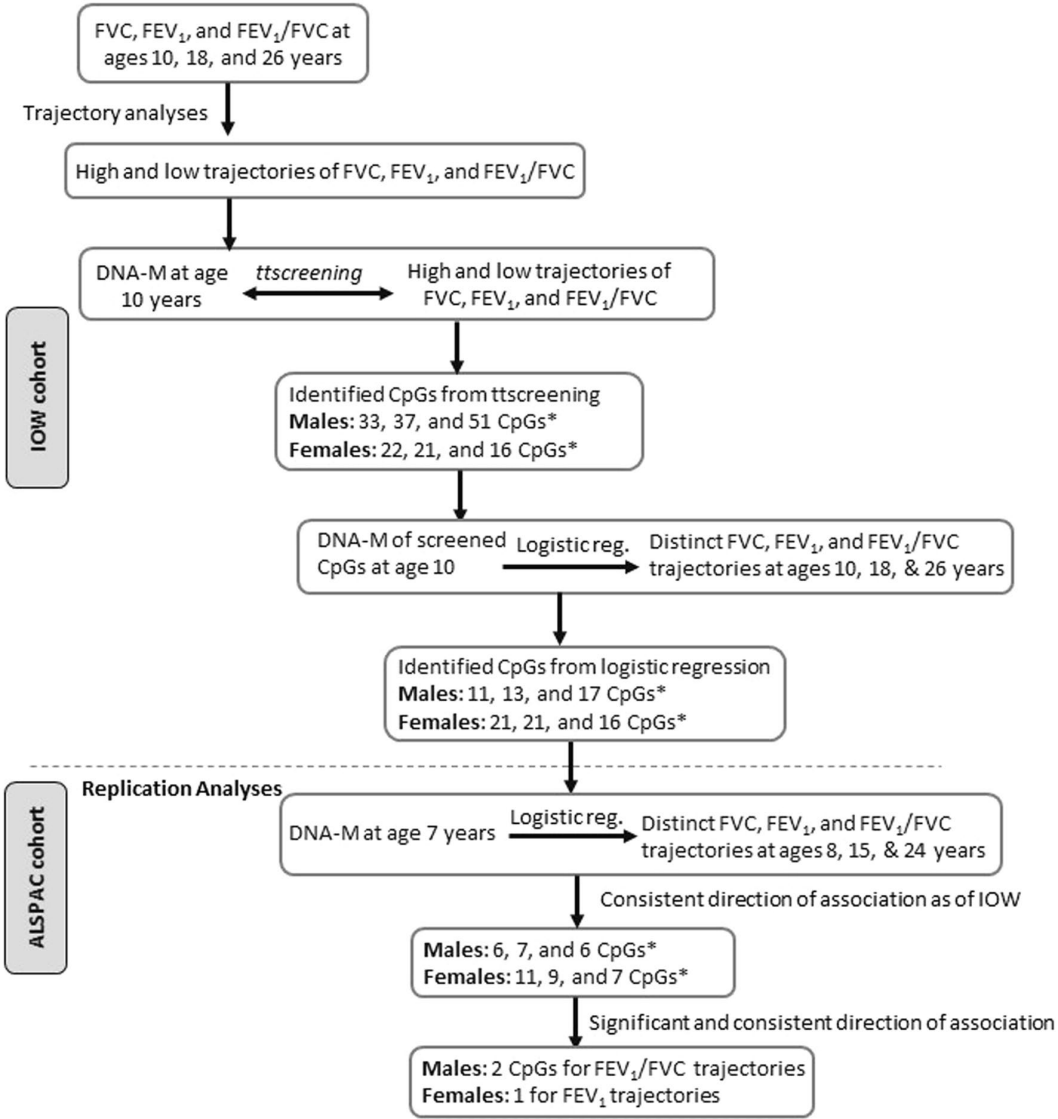
Flow chart of statistical analyses with the number of identified CpGs at each step. *Note*: *Number of significant CpGs were mentioned in an order for FVC, FEV_1_, and FEV_1_/ FVC changes, respectively

In total, 176 of the 377 male and 136 of the 432 female participants included in the analyses had DNA-M data available at age 10 years (Fig. 1). In screening, 119 distinct CpGs for males (33 CpGs for FVC, 37 for FEV_1_, and 51 for FEV_1_/FVC) and 56 distinct CpGs for females (22 CpGs for FVC, 21 for FEV_1_, and 16 for FEV_1_/FVC, Fig. 3) passed and were included in final analyses for their associations with lung function trajectories, with the effects of confounders adjustment. There was no overlap between the 119 and 56 CpGs identified in males and females, respectively.

Using multivariable logistic regression models, DNA-M levels at age 10 years of 11, 13, and 17 CpGs in males, and 21, 21, and 16 CpGs in females were statistically significantly associated with FVC, FEV_1_, and FEV_1_/FVC trajectories from pre-adolescence to adulthood, respectively, after correcting for multiple testing using the Bonferroni approach. Among the 96 distinct CpGs identified in the IOW cohort, 95 were further examined in the ALSPAC (cg24000797 was excluded during quality control in ALSPAC).

### Testing IOW cohort findings in the ALSPAC

In total, 4,861 participants (males = 2216) in ALSPAC had FVC, FEV_1_, and FEV_1_/FVC measurements for more than a single time point at ages 8-, 15-, and 24-year follow-up and were included in the trajectory analyses. Of these participants, 691 had DNA-M data at age 7 years.

**Fig. 4:**
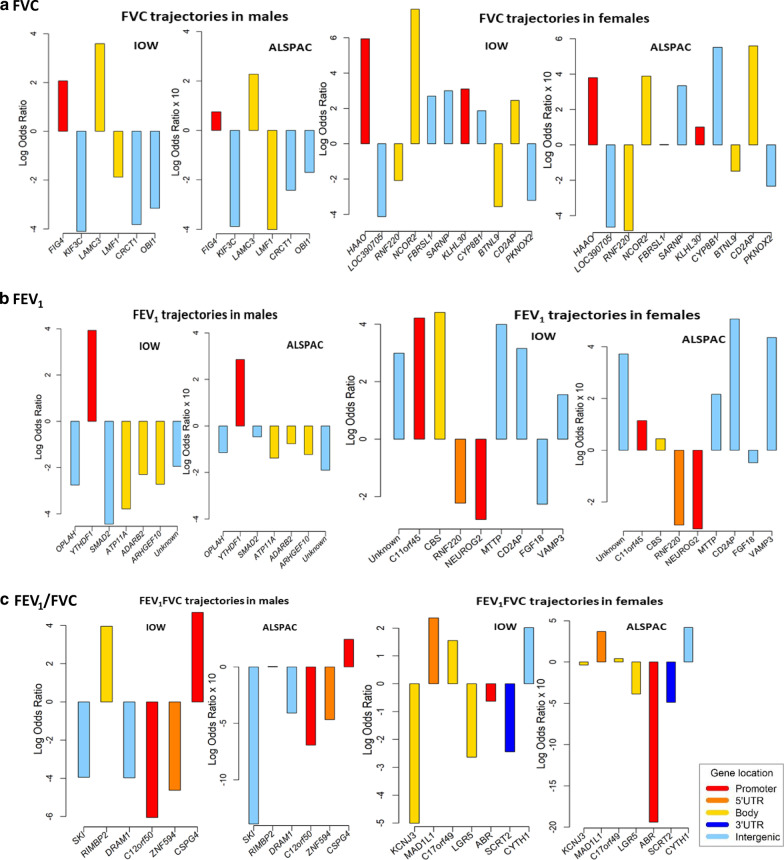
Bar plots of log odds ratios (*ORs) of IOW-ASPAC consistent CpGs and their mapped genes. *Note*: Each box plot showed CpGs which showed consistent associations between the IOW and ALSPAC cohorts for the association of DNA-M at childhood with lung function trajectories from childhood to adulthood in males and females, separately. *In ALSPAC the log odds ratios were smaller than the IOW cohort. For better visualization of bars in the ALSPAC figure, the log ORs were multiplied by 10

We identified two trajectories, low and high, for FVC, FEV_1_, and FEV_1_/FVC across 8, 15, and 24 years, comparable to those from the IOW cohort (Fig. 2). Next, for the 95 CpGs identified in the IOW cohort, we tested the association of DNA-M at age 7 years with lung function trajectories. Among the 95 CpGs, DNA-M at 44 distinct CpGs (2 CpGs overlapped between FEV_1_ and FVC in females) showed the consistent association with lung function trajectories with those in the IOW cohort in terms of the direction of association. These include 19 CpGs (6 CpGs for FVC, 7 for FEV_1_, and 6 for FEV_1_/FVC) in males, and 25 CpGs (11 CpGs for FVC, 9 for FEV_1_, and 7 for FEV_1_/FVC) in females (Table 2, Fig. 4, Additional file 1: Table S1). These 44 CpGs were noted as IOW-ALSPAC consistent CpGs. Among these CpGs, cg14669749 and cg21131402 showed statistically significant associations with FEV_1_/FVC trajectories in males and cg23987789 with FEV_1_ trajectories in females. DNA-M at three CpGs showed marginal statistical significance in females, two for FEV_1_/FVC trajectories (cg23190164, *p* = 0.08 and cg24479027, *p* = 0.09) and one for FVC (cg05597624, *p* = 0.088). At 74% of the 19 identified CpGs in males, a higher DNA-M was associated with a higher odds of being in the low lung function trajectories, while in females, the percentage was 44%.

**Table 2:** CpGs showing consistent associations of DNA-M with lung function trajectories between IOW cohort and ALSPAC

CpGs	Chr. no	Gene name	Location^a^	IOW cohort			ALSPAC cohort	
				Log ORs (95% CIs)	*P*_*Raw*_ value	*P*_*Bonferroni*_ value	Log ORs (95% CIs)	*P* value
Males								
FVC trajectories								
cg02304879	6	*FIG4*	Promoter	2.07 (1.02, 3.12)	0.0001	0.0034	0.07 (− 0.34, 0.49)	0.7251
cg02641801	2	*KIF3C*	Intergenic	− 4.1 (− 6.63, − 1.58)	0.0015	0.0350	− 0.39 (− 1.04, 0.26)	0.2417
cg04901044	9	*LAMC3*	Body	3.59 (1.72, 5.47)	0.0002	0.0051	0.23 (− 0.28, 0.73)	0.3756
cg16709691	16	*LMF1*	Body	− 1.87 (− 3.05, − 0.7)	0.0017	0.0402	− 0.40 (− 0.89, 0.08)	0.1056
cg23254163	1	*CRCT1*	Intergenic	− 3.82 (− 5.55, − 2.09)	0.0000	0.0005	− 0.24 (− 0.74, 0.25)	0.3370
cg26856578	13	*OBI1*	Intergenic	− 3.16 (− 4.87, − 1.44)	0.0003	0.0085	− 0.17(− 0.66, 0.32)	0.4992
FEV_1_ trajectories								
cg06319475	8	*OPLAH*	Intergenic	− 2.75 (− 4.41, − 1.09)	0.0012	0.0362	− 0.11 (− 0.59, 0.36)	0.6370
cg10605442	20	*YTHDF1*	Promoter	3.93 (1.56, 6.30)	0.0012	0.0362	0.28 (− 0.70, 1.27)	0.5702
cg12655437	18	*SMAD2*	Intergenic	− 4.44 (− 6.83, − 2.06)	0.0003	0.0092	− 0.05 (− 0.61, 0.51)	0.8713
cg19957503	13	*ATP11A*	Body	− 3.78 (− 6.03, − 1.53)	0.0010	0.0313	− 0.14 (− 0.57, 0.30)	0.5342
cg23131974	10	*ADARB2*	Body	− 2.3 (− 3.72, − 0.88)	0.0015	0.0384	− 0.08 (− 0.46, 0.30)	0.6945
cg24352757	8	*ARHGEF10*	Body	− 2.72 (− 4.19, − 1.24)	0.0003	0.0111	− 0.12 (− 0.60, 0.36)	0.6151
cg26844180	16	*Unknown*	Intergenic	− 1.94 (− 3.12, − 0.76)	0.0013	0.0364	− 0.19 (− 0.50, 0.12)	0.2296
FEV_1_/FVC trajectories								
**cg14669749**	**1**	***SKI***	**Intergenic**	**− 3.94 (− 6.21, − 1.67)**	**0.0007**	**0.0260**	**− 1.39 (− 2.72,− 0.07)**	**0.0397**
cg18499321	12	*RIMBP2*	Body	3.96 (2.11, 5.80)	0.0000	0.0014	0.005 (− 0.45, 0.46)	0.9843
cg21049825	12	*DRAM1*	Intergenic	− 3.98 (6.28, − 1.67)	0.0007	0.0269	− 0.41 (− 1.18, 0.36)	0.2992
**cg21131402**	**12**	***C12orf50***	**Promoter**	**− 6.05 (− 9.41, − 2.7)**	**0.0004**	**0.0173**	**− 0.69 (− 1.41, 0.02)**	**0.0357**
cg22904752	17	*ZNF594*	5′UTR	− 4.63 (− 7.25, − 2.01)	0.0005	0.0220	− 0.47 (− 1.43,0.50)	0.3449
cg27378180	15	*CSPG4*	Promoter	4.69 (2.02, 7.36)	0.0006	0.0233	0.24 (− 0.51, 1.00)	0.5247
Females								
FVC trajectories								
cg00081919	2	*HAAO*	Intergenic	5.94 (2.02, 9.86)	0.0030	0.0238	0.38 (− 0.60, 1.35)	0.4454
cg00514514	16	*LOC390705*	Intergenic	− 4.12 (− 6.68, − 1.56)	0.0016	0.0160	− 0.46 (− 1.14, 0.21)	0.1788
**cg05597624**	**1**	***RNF220***	**5′UTR**	**− 2.07 (− 3.63, − 0.52)**	**0.0089**	**0.0356**	**− 0.48 (− 1.04, 0.07)**	**0.0881**
cg06942010	12	*NCOR2*	Body	7.61 (3.90, 11.32)	0.0001	0.0012	0.39 (− 0.20, 0.98)	0.1968
cg07562175	12	*FBRSL1*	Intergenic	2.69 (1.08, 4.30)	0.0010	0.0156	0.002 (− 0.48, 0.48)	0.9936
cg12799537	12	*SARNP*	Body	3.00 (1.35, 4.66)	0.0004	0.0066	0.33 (− 0.27, 0.94)	0.2801
cg13168117	2	*KLHL30*	5′UTR	3.10 (1.24, 4.95)	0.0011	0.0156	0.10 (− 0.4, 0.60)	0.6915
cg13531735	3	*CYP8B1*	Intergenic	1.87 (0.28, 3.47)	0.0215	0.0431	0.55 (− 0.13, 1.23)	0.1135
cg16049690	5	*BTNL9*	Body	− 3.55 (− 5.19, − 1.92)	0.0000	0.0005	− 0.15 (− 0.43, 0.13)	0.3001
cg18876084	6	*CD2AP*	Intergenic	2.46 (0.84, 4.08)	0.0030	0.0238	0.56 (− 0.15, 1.27)	0.1235
cg22777186	11	*PKNOX2*	5′UTR	− 3.2 (− 5.1, − 1.30)	0.0010	0.0156	− 0.23 (− 0.99, 0.52)	0.5463
FEV_1_ trajectories								
cg00529742	1	*Unknown*	Intergenic	2.99 (1.22, 4.77)	0.0009	0.0160	0.37 (− 0.20, 0.94)	0.2004
cg04843085	11	*C11orf45*,* KCNJ5*	Promoter, 5′UTR	4.21 (1.13, 7.30)	0.0075	0.0470	0.11 (− 0.46, 0.69)	0.6961
cg05299847	21	*CBS*	Body	4.41 (2.27, 6.55)	0.0001	0.0011	0.04 (− 0.33, 0.42)	0.8174
cg05597624	1	*RNF220*	5′UTR	− 2.23 (− 3.82, − 0.65)	0.0058	0.0470	− 0.29 (− 0.8, 0.22)	0.2653
cg09707262	4	*NEUROG2*	Promoter	− 2.80 (− 4.70, − 0.90)	0.0038	0.0456	− 0.30 (− 1.1, 0.56)	0.4881
cg11479221	4	*MTTP*	Intergenic	4.00 (1.47, 6.52)	0.0019	0.0286	0.22 (− 0.43, 0.87)	0.5149
cg18876084	6	*CD2AP*	Intergenic	3.15 (1.39, 4.91)	0.0005	0.0086	0.51 (− 0.21, 1.22)	0.1658
cg22697108	5	*FGF18*	Intergenic	− 2.27 (− 4.04, − 0.49)	0.0125	0.0470	− 0.05 (− 0.5, 0.43)	0.8442
**cg23987789**	**1**	***VAMP3***	**Intergenic**	**1.55 (0.46, 2.63)**	**0.0052**	**0.0470**	**0.44 (0.09, 0.79)**	**0.0148**
FEV_1_/FVC trajectories								
cg03861217	2	*KCNJ3*	Body	− 5.00 (− 8.17, − 1.83)	0.0020	0.0142	− 0.04 (− 0.89, 0.82)	0.9356
cg19723734	7	*MAD1L1*	5′UTR	2.36 (0.48, 4.24)	0.0139	0.0417	0.37 (− 0.33, 1.07)	0.2985
cg20805367	17	*C17orf49*	Body	1.55 (0.63, 2.48)	0.0010	0.0096	0.04 (− 0.26, 0.34)	0.7861
**cg23190164**	**12**	***LGR5***	**Body**	**− 2.64 (− 4.31, − 0.97)**	**0.0020**	**0.0142**	**− 0.39 (− 0.83, 0.06)**	**0.0901**
**cg24479027**	**17**	***ABR***	**Promoter**	**− 0.63 (− 1.14, − 0.11)**	**0.0182**	**0.0417**	**− 1.94 (− 4.11, 0.24)**	**0.0807**
cg24595510	20	*SCRT2*	3′UTR	− 2.44 (− 4.07, − 0.82)	0.0032	0.0161	− 0.49 (− 1.51, 0.53)	0.3487
cg25729401	17	*CYTH1*	Intergenic	2.02 (0.28, 3.75)	0.0229	0.0417	0.42 (− 0.48, 1.32)	0.3614

log odds ratios (ORs) of IOW-ALSPAC consistent CpGs for the association of DNA-M at childhood with lung function trajectories from childhood-to-adulthood in males and females, separately

The CpGs showed the same direction of associations and were significant at 0.05 or 0.1 level and were in bold font

*Chr. no* chromosome number, *ORs* odds ratios, *CIs* confidence intervals

^a^Genes located at intergenic location were not found in Illumina annotation file and were identified using online tool SNIPPER

### Association with gene expression

In a time-lagged assessment of DNA-M at age 10 years with gene expression at 26 years (*n* = 35 males and 41 females), 15 of the 19 identified CpGs in males and another 15 CpGs of the 25 identified in females had expression data on the CpGs mapped genes. In males, of the 15 CpGs, DNA-M at cg16709691 (*LMF1*) and cg12655437 (*SMAD2*) was associated with the expression of genes (Table 3; both *p* values < 0.03). Among the 15 CpGs in females, DNA-M at cg16049690 (*BTNL9*), cg07562175 (*FBRSL1*), cg13168117 (*KLHL30*), and cg23987789 (*VAMP3*) was associated with gene expression (Table 3; all *p* values < 0.05). At these six CpGs, higher DNA-M at cg16709691 (*LMF1*) and cg16049690 (*BTNL9*) was associated with lower gene expression, while higher DNA-M at cg12655437 (*SMAD2*), cg07562175 (*FBRSL1*), cg13168117 (*KLHL30*), and cg23987789 (*VAMP3*) was associated with higher gene expression (Table 3).

**Table 3:** Association of DNA-M at IOW-ALSPAC consistent CpGs with gene expression

CpGs name	Gene name	Chr. no	Location^a^	DNA-M at age 10 years with gene expression at age 26 years	
				Coeff	*P* value
Males					
cg23131974	*ADARB2*	10	Body	0.004	0.902
cg24352757	*ARHGEF10*	8	Body	− 0.095	0.115
cg19957503	*ATP11A*	13	Body	0.017	0.729
cg27378180	*CSPG4*	15	Promoter	− 0.002	0.982
cg21049825	*DRAM1*	12	Intergenic	− 0.071	0.329
cg02304879	*FIG4*	6	Promoter	− 0.341	0.456
cg02641801	*KIF3C*	2	Intergenic	− 0.018	0.881
cg04901044	*LAMC3*	9	Body	− 0.037	0.571
**cg16709691**	***LMF1***	16	Body	**− 0.231**	**0.022**
cg06319475	*OPLAH*	8	Intergenic	0.002	0.980
cg18499321	*RIMBP2*	12	Body	− 0.070	0.186
cg14669749	*SKI*	1	Intergenic	0.009	0.909
**cg12655437**	***SMAD2***	**18**	**Intergenic**	**0.199**	**0.024**
cg10605442	*YTHDF1*	20	Promoter	− 0.004	0.976
cg22904752	*ZNF594*	17	5′UTR	0.022	0.648
Females					
cg24479027	*ABR*	17	Promoter	-0.048	0.925
**cg16049690**	***BTNL9***	**5**	**Body**	**− 0.102**	**0.034**
cg04843085	*C11orf45*	11	Promoter, 5′UTR	0.004	0.906
cg05299847	*CBS*	21	Body	− 0.030	0.536
cg18876084	*CD2AP*	6	Intergenic	− 0.024	0.665
cg25729401	*CYTH1*	17	Intergenic	− 0.265	0.165
**cg07562175**	***FBRSL1***	12	Intergenic	**0.304**	**0.045**
cg00081919	*HAAO*	2	Intergenic	− 0.087	0.186
**cg13168117**	***KLHL30***	2	5′UTR	**0.148**	**0.0043**
cg19723734	*MAD1L1*	7	5′UTR	0.234	0.085
cg06942010	*NCOR2*	12	Body	0.062	0.465
cg05597624	*RNF220*	1	5′UTR	− 0.096	0.701
cg12799537	*SARNP*	12	Body	0.058	0.462
**cg23987789**	***VAMP3***	1	Intergenic	**0.846**	**0.0285**
cg04843085	*KCNJ5*	11	Promoter, 5′UTR	− 0.100	0.735

The CpGs which showed a significant association with gene expression at 0.05 level were in bold font

^a^Genes located at intergenic location were not found in Illumina annotation file and were identified using online tool SNIPPER

*Chr. no* chromosome number, *coeff.* coefficient

### Detection of differentially methylated regions

For DMR enrichment analysis, a frequency of 20 and above was focused in screening to secure a sufficient number of CpGs. In females, 486, 518, and 461 CpGs and in males, 419, 559, and 842 CpGs for FVC, FEV_1_, and FEV_1_/FVC trajectory, respectively, were selected after screening and included in DMR analyses for each trajectory. After controlling FDR of 0.05, 23 statistically significant DMRs in males and 10 in females were identified (Table 4 with breakdown for each lung function trajectory). In total, 78 CpGs were in the 33 (23 + 10) identified DMRs (Additional file 1: Table S2), of which two CpGs (cg09707262 and cg02304879) were also among the 44 CpGs individually identified CpGs. The CpGs in the identified DMRs with the mapped genes and chromosomes, and the corresponding *p* values of DMRs for each sex are presented in Additional file 1: Figure S1.

**Table 4:** DMRs for lung function trajectory in relation to childhood DNA-M identified by DMRcate method

Lung function trajectory	Molecular location of the DMR (chromosome: start–end)	Annotated Genes	No. CpGs in the region^b^	Stouffer
Males				
FVC				
	chr6: 110011156–110011999	*FIG4*,* AK9*	2	1.15 × 10^–177^
	chr13: 112986154–112986635	*LINC01044*	4	9.26 × 10^–143^
	chr11: 113660695–113660756	*ATF4P4*,* RP11-667M19.5*	2	1.13 × 10^–113^
	chr1: 46859774–46859791	*FAAH*	2	3.51 × 10^–65^
FEV_1_				
	chr17: 28928406–28928453	*SMURF2P1*	2	1.24 × 10^–243^
	chr2: 172974138–172974630	*DLX2*^a^	2	1.14 × 10^–175^
	chr16: 31147044–31147177	*PRSS8*	2	1.67 × 10^–55^
	chr13: 37249426–37249450	*SERTM1*	2	1.78 × 10^–48^
	chr6: 31148383–31148552	*POU5F1*	6	3.27 × 10^–42^
	chr6: 74009041–74009455	*KHDC1*^a^	2	4.35 × 10^–14^
FEV_1_/FVC				
	chr2: 20424423–20425395	*SDC1*	2	1.03 × 10^–280^
	chr16: 84029457–84029584	NECAB2^a^	2	4.14 × 10^–235^
	chr22: 39713008–39713062	*RPL3*	2	1.07 × 10^–140^
	chr5: 177366867–177367013	*RP11-1252I4.2*	2	1.38 × 10^–100^
	chr1: 47656137–47656140	*PDZK1IP1*	2	3.78 × 10^–100^
	chr1: 223899845–223899998	*CAPN2*	2	1.41 × 10^–95^
	chr7: 1003645–1004748	*COX19*^a^	3	3.69 × 10^–79^
	chr6: 31846996–31847009	*SLC44A4*	2	3.88 × 10^–43^
	chr1: 11761078–11761296	DRAXIN^a^	2	3.60 × 10^–29^
	chr5: 23951555–23951696	*C5orf17*	2	8.99 × 10^–26^
	chr17: 78865368–78865662	*RPTOR*^a^	2	1.76 × 10^–15^
	chr15: 99409360–99409506	*NA*	2	1.12 × 10^–12^
	chr2: 65593908–65594021	*SPRED2*	2	1.99 × 10^–7^
Females				
FVC	chr1: 1022530–1022900	*C1orf159*	2	1.51 × 10^–139^
	chr11: 68621969–68622177	*CPT1A*^a^	2	3.25 × 10^–133^
	chr11: 68658383–68658836	*MRPL21*^a^	3	4.99 × 10^–7^
FEV_1_				
	chr4: 113437801–113438462	*NEUROG2*,* RP11-402J6.1*	2	6.18 × 10^–158^
	chr1: 7842369–7842406	*PER3-003*	2	1.33 × 10^–142^
	chr8: 144416404–144416485	*TOP1MT*	2	1.13 × 10^–115^
FEV_1_/FVC				
	chr16: 56715756–56716182	*MT1X*,* RP11-343H19.2*	2	1.73 × 10^–101^
	chr17: 79905219–79905255	*MYADML2*	2	1.69 × 10^–123^
	chr7: 73256414–73256416	*WBSCR27*	2	1.91 × 10^–17^
	chr20: 3051954–3052221	*OXT*	2	1.43 × 10^–5^

DMRcate annotates to UCSC RefGene from the Illumina annotation file

^a^Genes were not found in Illumina annotation file and were identified using online tool SNIPPER

^b^CpGs names are provided in Additional file 1: Table S2

### Biological pathways of mapped genes

The 44 IOW-ALSPAC consistent CpGs (19 CpGs for males and 25 for females) and the 78 CpGs in the 33 identified DMRs (23 DMRs in males and 10 in females) were mapped to 42 (Table 2) and 33 genes (Table 4), respectively. The distinct 73 genes were included in pathway enrichment analysis (focusing on pathways with at most 2000 genes) via bioinformatics tool ToppFun. After controlling the FDR of 0.05, six and 12 pathways were identified (Table 5) in males and females, respectively.

**Table 5:** Pathways were identified from the mapped genes of the IOW-ALSPAC consistent CpGs and DMRs

Name of the pathways	Source	*P* value	*q* value FDR B&H	Hit in query list
Males				
Defective B4GALT7 causes EDS, progeroid type	REACTOME	4.12 × 10^–4^	3.40 × 10^–2^	*SDC1*,* CSPG4*
Defective B3GALT6 causes EDSP2 and SEMDJL1	REACTOME	4.12 × 10^–4^	3.40 × 10^–2^	*SDC1*,* CSPG4*
Defective B3GAT3 causes JDSSDHD	REACTOME	4.12 × 10^–4^	3.40 × 10^–2^	*SDC1*,* CSPG4*
Downregulation of SMAD2/3: SMAD4 transcriptional activity	REACTOME	6.08 × 10^–4^	3.40 × 10^–2^	*SDC1*,* CSPG4*
A tetrasaccharide linker sequence is required for GAG synthesis	REACTOME	7.79 × 10^–4^	3.40 × 10^–2^	*SDC1*,* CSPG4*
Diseases associated with glycosaminoglycan metabolism	REACTOME	7.79 × 10^–4^	3.40 × 10^–2^	*SDC1*,* CSPG4*
Females				
Circadian entrainment	KEGG	6.15 × 10^–4^	2.86 × 10^–2^	*PER3*,* KCNJ3*,* KCNJ5*
Oxytocin signalling pathway	KEGG	2.36 × 10^–4^	3.15 × 10^–2^	*OXT*,* KCNJ3*,* KCNJ5*
Inhibition of voltage-gated Ca^2+^ channels via Gbeta/gamma subunits	REACTOME	8.73 × 10^–4^	2.86 × 10^–2^	*KCNJ3*,* KCNJ5*
G protein-gated Potassium channels	REACTOME	8.73 × 10^–4^	2.86 × 10^–2^	*KCNJ3*,* KCNJ5*
Activation of G protein-gated Potassium channels	REACTOME	8.73 × 10^–4^	2.86 × 10^–2^	*KCNJ3*,* KCNJ5*
Inwardly rectifying K + channels	REACTOME	1.34 × 10^–3^	2.86 × 10^–2^	*KCNJ3*,* KCNJ5*
GABA B receptor activation	REACTOME	2.23 × 10^–3^	3.09 × 10^–2^	*KCNJ3*,* KCNJ5*
Activation of GABAB receptors	REACTOME	2.23 × 10^–3^	3.09 × 10^–2^	*KCNJ3*,* KCNJ5*
Sterols are 12-hydroxylated by CYP8B1	REACTOME	3.53 × 10^–3^	4.00 × 10^–2^	*CYP8B1*
Amplification of signal from unattached kinetochores via a MAD2 inhibitory signal	REACTOME	3.53 × 10^–3^	4.00 × 10^–2^	*MAD1L1*
Amplification of signal from the kinetochores	REACTOME	3.53 × 10^–3^	4.00 × 10^–2^	*MAD1L1*
GABA receptor activation	REACTOME	4.33 × 10^–3^	4.66 × 10^–2^	*KCNJ3*,* KCNJ5*

## Discussion

Two major distinct lung function trajectories from pre-adolescence to adulthood in each sex were identified using latent class trajectory analyses in two population-based birth cohort. We showed that pre-adolescence DNA-M at 44 CpGs was associated with the trajectories. These CpGs mapped to 42 genes, which were enriched in 18 KEGG and REACTOME pathways. We identified 23 and 10 DMRs associated with the lung function trajectories in males and females, respectively. We further evaluated the functional effects of the identified CpGs by integrating gene expression. DNA-M at two CpGs in males and four in females at age 10 years was longitudinally associated with gene expression at age 26 year among the distinct set of 15 CpGs in each sex. Since in our study lung function trajectories cover from pre-adolescence transition till adulthood, and DNA-M was measured at an age close to the transition rather than a number of years before adolescence, the identified 44 CpG sites have a strong potential of high sensitivity to predict an individual’s lung function development from pre-adolescence to young adulthood.

The high and low trajectories of lung function identified in this study were the same as in our previous study [12] except that in this study subjects who had lung function measurement only at one-time point were excluded to improve the accuracy of trajectory assignment. The identified trajectories were also consistent with the main two trajectories of the previous reports from population-based studies including ours [4, 10, 12]. In a large-scale study, Belgrave et al. [10] (age 5–24 years) identified 4 distinct trajectories of FEV_1_; persistently high; normal; below average; and persistently low. In our study, on the other hand, two (high and low) trajectories of FVC, FEV_1_, and FEV_1_/FVC represented the data the best, which was likely due to the relatively smaller sample size. Moreover, like Belgrave et al. study, in this study, participants in the low FVC and FEV_1_ trajectory group did not achieve maximally attainable FVC and FEV_1_ and in the low FEV_1_/FVC trajectory group showed an accelerated decline from age 10 to 26 years (15% and 11% decline in males and females, respectively), compared to the declines in the high trajectory (9% and 10%, respectively) (Fig. 2), suggesting a risk of future COPD. The observations in this study also support the findings in the previous longitudinal studies by Lange et al. and Bui et al. [6, 11] that the persistently low lung function trajectory is associated with the risk of COPD in adults.

To our knowledge, this is the first study examining the association of pre-adolescence DNA-M with lung function trajectories ranging from pre-adolescence to post-adolescence. The identified CpGs and DMRs at childhood may provide insight into the pathogenesis of variations in lung function growth in adolescence. In addition, the associations of methylation at some identified CpGs with gene expression, such as cg16709691 (*LMF1*), cg12655437 (*SMAD2*), cg16049690 (*BTNL9*), cg07562175 (*FBRSL1*), cg13168117 (*KLHL30*), and cg23987789 (*VAMP3*) manifest the functional importance of the CpGs as biomarkers. Among these genes, *SMAD2*, *FBRSL1*, and *VAMP3* were associated with lung function, its related pathway, and COPD in previous studies [25–28]. Although most individually identified CpGs through logistic regressions were different from those in the DMRs due to different assumptions and statistical approaches between these two analyses, their mapped genes jointly involved at the biological pathways (Table 5).

Among the listed biological pathways linked to the mapped genes (Table 4), several pathways play a significant role in lung function and/or COPD, including downregulation of SMAD2/3: SMAD4 transcriptional activity, circadian entrainment, GABA B receptor activation, and activation of G protein-gated potassium channels [25, 29–32]. For example, downregulation of SMAD2/3: SMAD4 transcriptional activity plays a role in the regulation of TGF-β1-induced collagen expression in lung. Excessive collagen deposition is one of the characteristics of idiopathic pulmonary fibrosis that lead to impaired lung function later in life. The association of cg12655437 with *SMAD2* expression in this study revealed the pathway as functionally meaningful. Another pathway, the circadian rhythm regulates physiological diurnal variation of lung function through the autonomous peripheral circadian clock mechanisms. Clara cells in the bronchioles play a major role in such variations of lung function. These physiological oscillations are driven by transcriptional factors and genes such as *PER3*.

The CpGs showing consistent direction of associations with statistical significance at 0.05 or < 0.1 in both cohorts included cg14669749 (*SKI*), cg21131402 (*C12orf50*), cg23987789 (*VAMP3*), cg23190164 (*LGR5*), cg24479027 (*ABR*), and cg05597624 (*RNF220*). Some of these genes, such as *VAMP3*, *LGR5*, *PER3*, and *SDC1*, were found to be involved in the different physiological functions of lung and chronic lung disease [27, 28, 33–36]. Among these genes, *VAMP3* is found as one of the soluble *N*-ethylmaleimide-sensitive factor attachment protein receptors regulating mucin granule exocytosis. Mucin secretion is an innate immunity mechanism, which is harmfully upregulated in obstructive lung diseases including COPD [27, 28]. In addition, being in the intergenic region, the significant positive association of methylation at cg23987789 with the expression of *VAMP3* revealed a potential of this CpGs’ functionally regulatory role. *LGR5* is related to the WNT signalling cascades, which are the critical regulators of different developmental and pathophysiological processes in lung. Dysregulated *LGR5* expression influences to reduced WNT-β catenine signalling cascades, which is further linked to chronic lung disease including COPD [33, 34]. Among the annotated genes of the DMRs and also identified in circadian entrainment pathway, in females, *PER3* has been previously associated with childhood and adolescence lung function (FEV_1_) [35]; in males, *SDC1* was found as a differentially expressed genes in COPD development by robust rank aggregation method and in KEGG pathway in the previous study [36].

Although overall patterns of lung function trajectories in males and females were similar, for each identified trajectory, there existed large differences in volumes and flows between the two sex (Fig. 2). Such differences were expected and acknowledged in the literature [20–23] and were the major driving factors for the stratified study design. The uniqueness of identified CpGs for each sex led us to postulate the possibility of either different underlying epigenetic mechanisms in males and females in the regulation of gene activity and may act as the biomarkers of physiology and/or exposures that influence lung function trajectory. Another strength of this study was the time-lagged study design. Because of this, CpGs identified in this study have the potential to serve as candidate predictors for future lung function trajectories and will be beneficial to the detection of early lung diseases and subjects with a higher risk of developing those diseases.

Some issues related to the study designs and data analyses are worth discussing. In this study, participants with lung function measurements available only at one-time point were excluded from the analyses. The exclusion of subjects with missing data plus stratification by sex made the sample size smaller for each group, especially for females (*n* = 136). However, including participants with lung function measurement available at two or more time points ensured a high probability of trajectory group assignment rather random group assignment with a probability around 0.50. Also, based on the comparison with the whole study cohort, the study samples represented the whole cohort indicating that such restriction (≥ 2 repeated lung function measurements) did not bring statistically significant selection bias into the study samples. The use of a screening process, together with stringent control of multiple testing via the Bonferroni approach instead of controlling FDR, and the utilization of the replication cohort ensured that the findings from our study are robust with the potential of being generalized. Besides, in the IOW cohort, age 10 was treated as the pre-adolescence age, since almost all children (males 98% and females 92%) included in this study had not entered any phase of puberty. The children with minimal pubertal events were not excluded from the present study, since excluding them was not expected to alter the findings and conclusions but might have decreased testing power. Another perspective related to age is that, in the IOW cohort, the analyses were based on data collected at ages 10, 18, and 26 years representing pre- and post-adolescence. In the ALSPAC, the corresponding ages were 7/8, 15, and 24 years. The decline phase of lung function might have not started yet at age 24 years in the ALSPAC for some subjects (Fig. 3). This inconsistency between the two cohorts might be the cause of a lack of replication for some CpGs. Finally, the identified CpGs had minimal overlapping among FVC, FEV_1_, and FEV_1_/FVC trajectories, although in females, some mapped genes of identified CpGs associated with FVC, FEV_1_, and/or FEV_1_/FVC trajectories were involved in common pathways across these three different lung function parameters. Since DNA-M was measured in whole blood rather than in airways, although several studies support this non-invasive sampling approach of assessing DNA-M, the relevance of epigenetic changes measured in leukocytes in whole blood to gene expression in the lung remains unanswered and deserves further investigations of their biological evidence.

## Conclusion

Our study identified 44 CpGs with pre-adolescence DNA-M shown to be associated with lung function trajectories from pre-adolescence to young adulthood. These CpGs have a strong potential as candidate markers in future studies focusing on predicting an individual’s lung function trajectory. A well-designed study plan is warranted to comprehensively assess these CpGs’ joint contributions on lung function patterns.

## Methods

### Study subjects and design

#### The IOW cohort—Discovery cohort

The Isle of Wight (IOW) birth cohort is a population-based birth cohort established in 1989, UK. The study was originally approved by the IOW Local Research Ethics Committee at recruitment, and further assessments of this cohort are approved by the National Research Ethics Service, Committee South Central—Southampton B (06/Q1701/34). Informed written consent was obtained from participants or their parents before participating. The study enrolled 1456 eligible children of 1536 born between January 1989 and February 1990 (after exclusion of adoptions, infant deaths, and denial). Details of the IOW birth cohort of 1989 have been described elsewhere [37]. Longitudinal monitoring of allergic diseases, phenotypic measures, genetic, and assessments of environmental exposures were conducted at birth, ages 1, (94.4%), 2 (84.5%), 4 (83.6%), 10 (94.3%), 18 (90.2%), and 26 (70.9%) years.

#### Lung function

Pre-bronchodilator spirometric measurements, including forced vital capacity (FVC), forced expiratory volume in one second (FEV_1_), and the ratio of FEV_1_ over FVC (FEV_1_/FVC), were conducted at ages 10 (*n* = 980), 18 (*n* = 838), and 26 (*n* = 546) years and included in the study. FVC and FEV_1_ were measured using a Koko spirometer and software with a portable desktop device (both PDS Instrumentation, Louisville, KY, USA), and the FEV_1_/FVC ratio was calculated. Spirometry was conducted and evaluated according to the American Thoracic Society (ATS) guidelines [38, 39]. To conduct spirometry, participants were required to be free of respiratory infection for two weeks, not taking oral steroids, not taking any β-agonist for six hours and caffeine for at least 4 h.

#### Measurement of DNA methylation (DNA-M)

Peripheral blood samples collected at ages 10 years from *n* = 330 randomly selected subjects were used for DNA extraction via a standard salting out procedure [40]. DNA concentration was estimated by Qubit quantitation. For each sample, one-microgram DNA was bisulphite-treated for cytosine to thymine conversion using the EZ 96-DNA methylation kit (Zymo Research, Irvine, CA, USA), following the manufacturer’s protocol. DNA samples were randomly distributed on microarrays to control against batch effects. DNA-M was measured using HumanMethylation450K and HumanMethylationEPIC BeadChips (Illumina, Inc., SanDiego, CA, USA). Arrays were processed using a standard protocol as described elsewhere [41], with multiple identical control samples assigned to each bisulphite conversion batch to assess assay variability.

#### Preprocessing of DNA-M

Probes not reaching a detection *p* value of 10^–16^ in at least 95% of samples were excluded. The same criterion was applied to exclude samples, i.e. a sample with detection *p* value of > 10^–16^ in more than 5% of CpGs was excluded. CpGs on sex chromosomes were also excluded to avoid bias. DNA-M was then pre-processed using the “CPACOR” pipeline for data from both platforms [42]. DNA-M intensities were quantile normalized using the R computing package, *minfi* [43]. DNA-M *β* values for each CpG were then calculated as a ratio of methylated (*M*) over the sum of methylated and unmethylated (*U*) probes (*β* = M/[*c* + *M* + *U*]) interpreted as the percentage of methylation [44], where c was used as a constant to prevent zero in the denominator. Principal components (PCs) inferred based on control probes were used to represent latent variables explaining chip-to-chip and technical (batch) effects on DNA-M variations. Since DNA-M data were from two different platforms (450 k and EPIC), we determined the PCs based on DNA-M at shared control probes between the two platforms. In total, 195 control probes were overlapped between 220 control probes from the 450 K BeadChips and 204 from the EPIC array. These 195 shared probes were then used to calculate the control probe PCs, top 15 of which were used to represent latent batch factors [42].

After pre-processing, a total of 473,864 and 847,155 CpGs were available in the 450 K and EPIC methylation array data, respectively, and 439,635 overlapping CpGs were identified between the two platforms. CpGs with a single nucleotide polymorphisms (SNP) overlapping the detection probe with minor allele frequency ≥ 0.7% (corresponding to at least 10 subjects in the IOW cohort with *n* = 1456) within 10 base pairs of the targeted CpG were excluded due to potential bias that those SNPs brought to the measurement of DNA-M. After excluding probe SNPs, 402,714 CpGs were included in the statistical analyses.

### Gene expression data

RNA-seq gene expression data for subjects at age 26 years were available in IOW cohort, which was used to evaluate biological relevance of CpGs shown to have time-lagged associations with lung function. We used paired-end (2 × 75 bp) RNA sequencing with the Illumina Tru-Seq Stranded mRNA Library Preparation Kit with IDT for Illumina Unique Dual Index (UDI) barcode primers following manufacturer’s recommendations. RNA samples were extracted from whole blood of the IOW cohort participants at age 26 years. All samples were sequenced second time using the identical protocol, and for each sample, the output from both runs was combined. FASTQC were run to assess the quality of the FASTQ files [45]. Reads were mapped against Human Genome (GRch37 version 75) using HISAT2 (v2.1.0) aligner [46]. The alignment files, produced in the Sequence Alignment Map (SAM) format, were converted into the Binary Alignment Map (BAM) format using SAMtools (v1.3.1) [47]. HTseq (v0.11.1) was used to count the number of reads mapped to each gene in the same reference genome used for alignment [48]. Normalized read count FPKM (Fragments Per Kilobase of transcript per Million mapped reads) were calculated using the countToFPKM package (https://github.com/AAlhendi1707/countToFPKM) and were included for subsequent data for analysis.

#### Confounders

Based on prior knowledge in the published literature, variables potentially associated with lung function trajectories in addition to DNA-M were included in the model as confounders. The potential confounders were birth weight, gestational age, duration of breastfeeding, maternal smoking exposure during pregnancy, recurrent chest infection collected at ages 1/2 years, second-hand smoking exposure at age 10 years (childhood), height and body mass index (BMI) at age 10 years, exposure to pets at age 10 years, age of puberty onset, and socioeconomic status (SES) [4, 11, 12].

Gestational age information was recorded during delivery in the hospital. Birth weight was measured immediately after birth. Heights and weights at age 10 were measured before spirometric measurements, and BMI was calculated from height and weight accordingly. The age of puberty onset was estimated based on self-reports about age of initiation of five pubertal markers for each sex, a growth spurt, body hair growth, skin changes, deepening voice of male, facial hair of male, breast development of female, and initiation of menstruation of female. The National Institute of Child and Human Development questionnaire from the Study of Early Child Care and Youth Development was used to identify pubertal stages. Information on second-hand smoke exposure in childhood was collected from parents. SES was classified using the composite “SES-cluster” method based on the following three variables: (a) the British socioeconomic classes [1–6] derived from parental occupation reported at birth; (b) the number of children in the index child’s bedroom (collected at age 4 years); and (c) family income at age 10 years [49]. This composite variable captures the family social class across the entire study period. Pet exposure information was collected at age 10 years through questionnaires.

#### The ALSPAC cohort—Replication cohort

ALSPAC is a population-based birth cohort study established in 1991 in Avon, UK, approximately 75 miles from the IOW [50, 51]. All pregnant women who were expecting to deliver between 1 April 1991 and 31 December 1992, and residing in the Avon region of the South West of England were eligible to be recruited. In total, 14,541 pregnant women were recruited for the study, of those 13,761 were eligible with 10,321 having DNA sampled. Information on environment, lifestyle, and health of the child and family was collected through annual questionnaires since the child’s birth. At age 7 an additional 913 children were enrolled. The total sample size for analyses using any data collected after the age of 7 is therefore 15,454 pregnancies, resulting in 15,589 foetuses. Of these 14,901 were alive at 1 year of age. From age 7 years, the participants were invited to an annual research clinic to obtain the exposure and other demographic data annually. Spirometry (Vitalograph 2120; Vitalograph, Maids Moreton, UK) was performed at 8, 15, and 24 years of age according to ATS standards [39, 52, 53], the same method as that applied in the IOW cohort. The study website contains details of all the data that are available through a fully searchable data dictionary and variable search tool (http://www.bristol.ac.uk/alspac/researchers/our-data/).

DNA-M data of children at ages 7 (*n* = 968) years were included in the study. DNA-M in peripheral blood was assessed using the Infinium HumanMethylation450K BeadChip [54]. The procedure for DNA sample preparation was comparable to that applied in the IOW cohort. The pre-processing of DNA-M was performed by adjusting batch effect, excluding CpGs with detection *p* value ≥ 0.01, and excluding samples that were flagged a sex-mismatch based on X-chromosome methylation. Details of pre-processing, quality control, and quantile normalization of DNA-M data have been described elsewhere [54, 55].

### Statistical analyses

#### Descriptive analyses

To evaluate whether subjects included in the study reasonably represented those in the complete study cohort, we compared lung function tests at ages 10, 18, and 26 years in the study samples with those of the complete cohort using one-sample *t* tests.

#### Determining distinctive lung function trajectories

Our previous publication [12] of lung function trajectory was based on at least single spirometry test to attain a maximum sample size. In this study, subjects with at least two-time point tests were included for trajectory analyses to improve the average posterior probability and to avoid the random assignment of the subjects into a trajectory. An unsupervised semi-parametric mixture modelling implemented in the SAS procedure PROC TRAJ [56] was applied to identify developmental lung function trajectories of FVC, FEV_1_, and FEV_1_/FVC over time (10, 18, and 26 years) for males and females separately [57], the same approach applied in our previous study [12]. This method combines the latent growth curve and mixture modelling approaches to detect distinct groups of trajectories [56]. All possible models were evaluated each with different numbers of groups (i.e. 2, 3, and 4) and different shapes of the trajectories (linear, quadratic, and cubic) for each group. Trajectory parameters were estimated using the maximum likelihood approach [58, 59]. The best model was selected based on two criteria, being as parsimonious as possible to summarize the distinctive features and with high Bayesian information criterion (BIC) [57, 60, 61]. To improve the quality of identified trajectories, in addition to BIC, probability of trajectory assignment as well as sample sizes in each group was further incorporated; the average posterior probabilities of assignment to a group were set at least 0.7, and the sample size of each group was required to be at least 5% of the total sample size [60]. Individuals were assigned to one of the trajectories/groups based on their highest estimated group-membership probabilities. The assigned group (categorical variable) of distinct lung function trajectories was used in subsequent analyses.

#### Association analyses

To assess the association of DNA-M at an earlier age with lung function trajectories at later ages in the IOW cohort, we followed a two-step analytical approach. In the first step, CpGs were screened to exclude CpGs potentially not associated with lung function trajectories using *ttScreening* (R package 3.5.1 version) [62, 63]. This method utilizes training and testing data in robust linear regressions with surrogate variables included in the regressions to adjust for unknown effects. The training and testing steps were repeated 100 times. A CpG that was statistically significant in both training and testing steps at least 50 times was included in the final set for subsequent regression analyses. The screening was performed for each lung function parameter, stratified by sex.

In second step, CpGs that passed screening were further assessed in logistic regression models in SAS 9.4 for the trajectories of each lung function parameter stratified by sex and adjusting for the above-mentioned confounders. Lung function trajectory was treated as the outcome variable, and the DNA-M at each CpG that passed screening was used as an independent variable. Multiple testing was corrected using the Bonferroni method with an experiment-wise significance level of 0.05. In all analyses, DNA-M adjusted for cell types, principle components, and batch effects at each CpG was used.

#### Replication analysis—in the ALSPAC cohort

The identified CpGs from the IOW cohort were further examined in the ALSPAC. Comparable analytical methods as those implemented in the IOW cohort were applied except for the exclusion of two covariates in regression analyses, recurrent chest infection, and pet exposure at childhood, which were unavailable in the ALSPAC.

#### Gene expression analysis

To assess the potential biological relevance of the identified CpGs, we examined the time-lagged association of DNA-M at those CpGs with the expression of their mapped genes. Linear regressions were applied to DNA-M at age 10 years with gene expression at age 26 years to each CpG which showed the consistent direction of association between the IOW cohort and ALSPAC.

#### Analyses of differentially methylated regions (DMRs)

To detect regional differential methylation signals among the CpGs that passed screening, an R package DMRcate was used [64]. The default settings in DMRcate include having at least two CpGs in a region and a minimum length of 1000 nucleotides. In our study, a DMR was considered to be statistically significant if the false discovery rate (FDR)-adjusted *p* value was < 0.05 [64]. Since DMR analyses focus on contribution of a region as a whole unit, a significant DMR can be detected even if there is no genome-wide significant individual CpGs in the region.

#### Pathway analyses

Genes annotated to the CpGs explored in ALSPAC with respect to the direction of odds ratios (ORs in the log scale) and DMRs were identified based on Illumina's manifestation file and SNIPPER (https://csg.sph.umich.edu/boehnke/snipper/) version 1.2. Bioinformatic assessment of the genes was conducted using the online bioinformatics tool ToppFun, available in the ToppGene Suite [65]. Multiple testing was adjusted by controlling FDR of 0.05.

## Supplementary Information

**Additional file 1.** **Table S1:** List of CpGs (k = 96) showing in both consistent and opposite direction of associations of DNA-M at childhood with lung function trajectories childhood-to-young adulthood in males and females between the IOW cohort and ALSPAC. **Table S2:** DMRs (k = 33) for lung function trajectory in relation to childhood DNA-M identified by DMRcate (FDR < 0.05) method. **Figure S1:** Circular plots of CpGs identified in DMRs (A) for males (B) for females.

## Data Availability

The datasets used and/or analysed during the current study are available from the corresponding author on reasonable request.

## Abbreviations

ATS: American Thoracic Society
ALSPAC: Avon Longitudinal Study of Parents and Children
BMI: Body mass index
CpGs: Cytosine-phosphate-guanine dinucleotide site or sites
COPD: Chronic obstructive pulmonary disease
DMR: Differentially methylated regions
DNA-M: DNA methylation
FVC: Forced vital capacity
FEV_1_: Forced expiratory volume in one second
FDR: False discovery rate
IOW: Isle of Wight
ORs: Odds ratios
PCs: Principal components
*ttScreening*: Training and testing screening
