# Supplementary material for: Pre-adolescence DNA methylation is associated with lung function trajectories from pre-adolescence to adulthood
Source: Clin Epigenetics. 2021 Jan 6;13:5. doi: 10.1186/s13148-020-00992-5 (PMC7789734; doi:10.1186/s13148-020-00992-5)
Supplement: Supplementary file 1 — Additional file 1. Table S1: List of CpGs (k = 96) showing in both consistent and opposite direction of associations of DNA-M at childhood with lung function trajectories childhood-to-young adulthood in males and females between the IOW cohort and ALSPAC. Table S2: DMRs (k = 33) for lung function trajectory in relation to childhood DNA-M identified by DMRcate (FDR < 0.05) method. Figure S1: Circular plots of CpGs identified in DMRs (A) for males (B) for females. [file 13148_2020_992_MOESM1_ESM.docx]

**Additional file 1**

**Title**: **Pre-adolescence DNA methylation is associated with lung function trajectories from pre-adolescence to adulthood**

**Authors list**

Shadia Khan Sunny^1^, MBBS, MPH, email: ssunny@memphis.edu

Hongmei Zhang^1^, PhD, email: [hzhang6@memphis.edu](mailto:hzhang6@memphis.edu)

Fawaz Mzayek^1^, MD, MPH, PhD, email: [fmzayek@memphis.edu](mailto:fmzayek@memphis.edu)

Caroline L. Relton^2^, PhD, email: [Caroline.Relton@bristol.ac.uk](mailto:Caroline.Relton@bristol.ac.uk)

Susan Ring^2,3^, PhD, email: email: S.M.Ring@bristol.ac.uk

A. John Henderson^3^, MD, email: hong_mei_zhang@hotmail.com

Susan Ewart^4^, PhD, email: ewarts@cvm.msu.edu

John W. Holloway^5,6^, PhD, email: [J.W.Holloway@soton.ac.uk](mailto:J.W.Holloway@soton.ac.uk)

S. Hasan Arshad^6,7,8^, MBBS, MRCP, email: S.H.Arshad@soton.ac.uk

^1^ Division of Epidemiology, Biostatistics, and Environmental Health, School of Public Health, University of Memphis, Memphis, TN, USA.

^2^ MRC Integrative Epidemiology Unit, University of Bristol, Bristol, BS8 2BN, UK.

^3^ Population Health Sciences, University of Bristol, Bristol, BS8 2BN, UK.

^4^ Large Animal Clinical Sciences, Michigan State University, East Lansing, MI.

^5^ Human Development and Health, Faculty of Medicine, University of Southampton, Southampton, SO16 6YD, UK.

^6^ NIHR Southampton Biomedical Research Centre, University Hospital Southampton, Southampton, SO16 6YD, UK.

^7^ Clinical and Experimental Sciences, Faculty of Medicine, University of Southampton, Southampton, SO16 6YD, UK.

^8^ The David Hide Asthma and Allergy Research Centre, St Mary’s Hospital, Parkhurst Road, Newport, Isle of Wight PO30 5TG, UK.

**Corresponding author:**

Hongmei Zhang, PhD

Division of Epidemiology, Biostatistics, and Environmental Health Sciences,

School of Public Health, University of Memphis, Memphis, TN 38152, U.S.A.

Email: hzhang6@memphis.edu

**Table S1: List of CpGs (k = 96) showing in both consistent and opposite direction of associations of DNA-M at childhood with lung function trajectories childhood-to-young adulthood in males and females between the IOW cohort and ALSPAC.**

| **Lung function trajectories** | **CpGs** | **Chr. no.** | **Gene Name** | **Location*** | **IOW cohort** | | | **ALSPAC cohort** | |
| --- | --- | --- | --- | --- | --- | --- | --- | --- | --- |
|  |  |  |  |  | **Log ORs (95% CIs)** | ***P_Raw_*** | ***P_Bonferroni_*** | **Log ORs (95% CIs)** | ***P*** |
| **Males** |  |  |  |  |  |  |  |  |  |
| **FVC** |  |  |  |  |  |  |  |  |  |
|  | cg02304879 | 6 | *AKD1; FIG4* | 5'UTR; Promoter | 2.07 (1.02, 3.12) | 0.0001 | 0.0034 | 0.07 (-0.34, 0.49) | 0.7251 |
|  | cg02641801 | 2 | *KIF3C* | Intergenic | -4.1 (-6.63, -1.58) | 0.0015 | 0.0350 | -0.39 (-1.04, 0.26) | 0.2417 |
|  | cg03298700 | 2 | *SPEG* | Intergenic | -4.26 (-6.34, -2.18) | 0.0001 | 0.0019 | 0.23 (-0.26, 0.72) | 0.3632 |
|  | cg04901044 | 9 | *LAMC3* | Body | 3.59 (1.72, 5.47) | 0.0002 | 0.0051 | 0.23 (-0.28, 0.73) | 0.3756 |
|  | cg14860255 | 4 | *PPAT* | 3'UTR | -2.67 (-4.29, -1.05) | 0.0012 | 0.0305 | 0.15 (-0.26, 0.57) | 0.4648 |
|  | cg16709691 | 16 | *LMF1* | Body | -1.87 (-3.05, -0.70) | 0.0017 | 0.0402 | -0.4 (-0.89, 0.08) | 0.1056 |
|  | cg20627652 | 17 | *YPEL2* | Intergenic | 4.25 (1.96, 6.54) | 0.0003 | 0.0079 | -0.17 (-0.62, 0.27) | 0.4516 |
|  | cg23254163 | 1 | *CRCT1* | Intergenic | -3.82 (-5.55, -2.09) | 0.0000 | 0.0005 | -0.24 (-0.74, 0.25) | 0.3370 |
|  | cg23907982 | 17 | *STX8* | Promoter | -3.34 (-5.24, -1.43) | 0.0006 | 0.0156 | 0.18 (-0.32, 0.68) | 0.4845 |
|  | cg26856578 | 13 | *OBI1* | Intergenic | -3.16 (-4.87, -1.44) | 0.0003 | 0.0085 | -0.17 (-0.66, 0.32) | 0.4992 |
| **FEV_1_** |  |  |  |  |  |  |  |  |  |
|  | cg02571503 | 1 | *CNST* | Body | -4.49 (-7.00, -1.97) | 0.0005 | 0.0156 | 0.29 (-0.19, 0.77) | 0.2302 |
|  | cg02720761 | 7 | *PTPRN2* | Body | 4.51 (2.23, 6.78) | 0.0001 | 0.0038 | -0.09 (-0.47, 0.29) | 0.6405 |
|  | cg06319475 | 8 | *OPLAH* | Intergenic | -2.75 (-4.41, -1.09) | 0.0012 | 0.0362 | -0.11 (-0.59, 0.36) | 0.6370 |
|  | cg07827435 | 20 | *COL9A3* | Promoter | -2.35 (-3.77, -0.92) | 0.0013 | 0.0364 | 0.02 (-0.40, 0.44) | 0.9313 |
|  | cg10605442 | 20 | *YTHDF1* | Promoter | 3.93 (1.56, 6.3) | 0.0012 | 0.0362 | 0.28 (-0.7, 1.27) | 0.5702 |
|  | cg12405121 | 7 | *MIR4283-1* | Intergenic | 2.89 (1.06, 4.72) | 0.0019 | 0.0486 | -0.35 (-0.84, 0.14) | 0.1586 |
|  | cg12655437 | 18 | *SMAD2* | Intergenic | -4.44 (-6.83, -2.06) | 0.0003 | 0.0092 | -0.05 (-0.61, 0.51) | 0.8713 |
|  | cg14503291 | 17 | *FAM117A* | Body | 2.81 (1.10, 4.52) | 0.0013 | 0.0364 | -0.64 (-1.67, 0.39) | 0.2245 |
|  | cg19957503 | 13 | *ATP11A* | Body | -3.78 (-6.03, -1.53) | 0.0010 | 0.0313 | -0.14 (-0.57, 0.3) | 0.5342 |
|  | cg20692730 | 17 | *ATP2A3* | Body | 2.99 (1.35, 4.63) | 0.0004 | 0.0120 | -0.26 (-0.63, 0.11) | 0.1638 |
|  | cg23131974 | 10 | *ADARB2* | Body | -2.30 (-3.72, -0.88) | 0.0015 | 0.0384 | -0.08 (-0.46, 0.3) | 0.6945 |
|  | cg24352757 | 8 | *ARHGEF10* | Body | -2.72 (-4.19, -1.24) | 0.0003 | 0.0111 | -0.12 (-0.60, 0.36) | 0.6151 |
|  | cg26844180 | 16 | *Not found* | Intergenic | -1.94 (-3.12, -0.76) | 0.0013 | 0.0364 | -0.19 (-0.50, 0.12) | 0.2296 |
| **FEV_1_/FVC** |  |  |  |  |  |  |  |  |  |
|  | cg00646396 | 1 | *FAM110D* | Intergenic | 5.49 (2.61, 8.37) | 0.0002 | 0.0088 | -0.38 (-1.6, 0.83) | 0.5345 |
|  | cg00812861 | 9 | *KIAA1539* | 5'UTR | 5.47 (2.34, 8.59) | 0.0006 | 0.0241 | -0.02 (-0.72, 0.68) | 0.9645 |
|  | cg01288184 | 18 | *CABLES1* | Body | 2.96 (1.47, 4.45) | 0.0001 | 0.0049 | -0.1 (-0.63, 0.42) | 0.7022 |
|  | cg02843500 | 16 | *C16orf58* | 3'UTR | 5.10 (2.30, 7.90) | 0.0004 | 0.0154 | -0.42 (-1.25, 0.4) | 0.3171 |
|  | cg08200625 | 19 | *F2RL3* | Body | 5.47 (2.61, 8.33) | 0.0002 | 0.0084 | -0.24 (-0.88, 0.39) | 0.4492 |
|  | cg08722675 | 4 | *ACOX3* | Body | 4.54 (2.22, 6.87) | 0.0001 | 0.0061 | -0.91 (-1.65, -0.17) | 0.0161 |
|  | cg09989251 | 14 | *SAMD4A* | Intergenic | -3.75 (-5.96, -1.55) | 0.0009 | 0.0319 | 0.11 (-0.68, 0.9) | 0.7871 |
|  | cg11885407 | 3 | *OPA1* | Promoter | -1.68 (-2.52, -0.84) | 0.0001 | 0.0049 | 0.54 (-0.64, 1.72) | 0.3681 |
|  | cg13615983 | 9 | *MAMDC4* | Promoter | -3.45 (-5.51, -1.39) | 0.0010 | 0.0374 | 0.16 (-0.47, 0.78) | 0.6259 |
|  | cg14669749 | 1 | *SKI* | Intergenic | -3.94 (-6.21, -1.67) | 0.0007 | 0.0260 | -1.39 (-2.72, -0.07) | 0.0397 |
|  | cg18039133 | 20 | *BMP2* | Body | 4.06 (1.60, 6.51) | 0.0012 | 0.0415 | -0.32 (-0.9, 0.26) | 0.2799 |
|  | cg18499321 | 12 | *RIMBP2* | Body | 3.96 (2.11, 5.80) | 0.0000 | 0.0014 | 0.01 (-0.45, 0.46) | 0.9843 |
|  | cg21049825 | 12 | *DRAM1* | Intergenic | -3.98 (-6.28, -1.67) | 0.0007 | 0.0269 | -0.41 (-1.18, 0.36) | 0.2992 |
|  | cg21131402 | 12 | *C12orf50* | Promoter | -6.05 (-9.41, -2.70) | 0.0004 | 0.0173 | -0.69 (-1.41, 0.02) | 0.0357 |
|  | cg22904752 | 17 | *ZNF594* | 5'UTR | -4.63 (-7.25, -2.01) | 0.0005 | 0.0220 | -0.47 (-1.43, 0.50) | 0.3449 |
|  | cg24754987 | 5 | *SPATA24* | Promoter | 3.41 (1.59, 5.24) | 0.0002 | 0.0112 | -0.86 (-2.57, 0.84) | 0.3208 |
|  | cg27378180 | 15 | *CSPG4* | Promoter | 4.69 (2.02, 7.36) | 0.0006 | 0.0233 | 0.24 (-0.51, 1.00) | 0.5247 |
| **Females** |  |  |  |  |  |  |  |  |  |
| **FVC** |  |  |  |  |  |  |  |  |  |
|  | cg00081919 | 2 | *HAAO* | Intergenic | 5.94 (2.02, 9.86) | 0.0030 | 0.0238 | 0.38 (-0.6, 1.35) | 0.4454 |
|  | cg00514514 | 16 | *LOC390705* | Intergenic | -4.12 (-6.68, -1.56) | 0.0016 | 0.0160 | -0.46 (-1.14, 0.21) | 0.1788 |
|  | cg00671878 | 7 | *HECW1* | 5'UTR | -4.24 (-6.84, -1.63) | 0.0014 | 0.0160 | 0.58 (0.11, 1.04) | 0.0147 |
|  | cg02528008 | 5 | *MCC* | Body | -2.36 (-4.12, -0.60) | 0.0086 | 0.0356 | 0.43 (-0.17, 1.03) | 0.1580 |
|  | cg02700891 | 3 | *TRH* | 5'UTR | 1.41 (0.44, 2.39) | 0.0045 | 0.0271 | -0.44 (-1.26, 0.39) | 0.3005 |
|  | cg05597624 | 1 | *RNF220* | 5'UTR | -2.07 (-3.63, -0.52) | 0.0089 | 0.0356 | -0.48 (-1.04, 0.07) | 0.0881 |
|  | cg06942010 | 12 | *NCOR2* | Body | 7.61 (3.90, 11.32) | 0.0001 | 0.0012 | 0.39 (-0.20, 0.98) | 0.1968 |
|  | cg07562175 | 12 | *FBRSL1* | Intergenic | 2.69 (1.08, 4.30) | 0.0010 | 0.0156 | 0.01 (-0.48, 0.48) | 0.9936 |
|  | cg07642566 | 19 | *MIR7-3* | Promoter | 7.11 (3.59, 10.63) | 0.0001 | 0.0015 | -0.61 (-1.43, 0.21) | 0.1472 |
|  | cg09143964 | 5 | *FAM196B* | 5'UTR | -3.34 (-5.15, -1.53) | 0.0003 | 0.0053 | 0.67 (-0.11, 1.46) | 0.0932 |
|  | cg11314787 | 6 | *MLN* | Intergenic | -3.95 (-6.40, -1.50) | 0.0016 | 0.0160 | 0.16 (-0.49, 0.82) | 0.6301 |
|  | cg11794398 | 6 | *MAP3K4* | Intergenic | -3.76 (-6.01, -1.51) | 0.0010 | 0.0156 | 0.29 (-0.61, 1.18) | 0.5308 |
|  | cg12799537 | 12 | *SARNP* | Body | 3.00 (1.35, 4.66) | 0.0004 | 0.0066 | 0.33 (-0.27, 0.94) | 0.2801 |
|  | cg13168117 | 2 | *KLHL30* | 5'UTR | 3.1 (1.24, 4.95) | 0.0011 | 0.0156 | 0.10 (-0.40, 0.60) | 0.6915 |
|  | cg13531735 | 3 | *CYP8B1* | Intergenic | 1.87 (0.28, 3.47) | 0.0215 | 0.0431 | 0.55 (-0.13, 1.23) | 0.1135 |
|  | cg15717637 | 5 | *CSNK1G3* | Promoter | -3.52 (-5.31, -1.73) | 0.0001 | 0.0021 | 0.21 (-0.29, 0.72) | 0.4044 |
|  | cg16049690 | 5 | *BTNL9* | Body | -3.55 (-5.19, -1.92) | 0.0000 | 0.0005 | -0.15 (-0.43, 0.13) | 0.3001 |
|  | cg17341904 | 12 | *NEDD1* | Intergenic | -2.33 (-4.02, -0.63) | 0.0071 | 0.0356 | 0.70 (0.10, 1.30) | 0.0215 |
|  | cg18876084 | 6 | *CD2AP* | Intergenic | 2.46 (0.84, 4.08) | 0.0030 | 0.0238 | 0.56 (-0.15, 1.27) | 0.1235 |
|  | cg22777186 | 11 | *PKNOX2* | 5'UTR | -3.20 (-5.10, -1.30) | 0.0010 | 0.0156 | -0.23 (-0.99, 0.52) | 0.5463 |
|  | cg22842048 | 11 | *MRGPRF* | Body | 3.21 (1.25, 5.17) | 0.0013 | 0.0160 | -0.49 (-1.09, 0.12) | 0.1148 |
| **FEV_1_** |  |  |  |  |  |  |  |  |  |
|  | cg00529742 | 1 | *Not found* | Intergenic | 2.99 (1.22, 4.77) | 0.0009 | 0.0160 | 0.37 (-0.20, 0.94) | 0.2004 |
|  | cg01830900 | 7 | *LOC389458* | Promoter | -2.88 (-4.65, -1.12) | 0.0013 | 0.0213 | 0.11 (-0.22, 0.44) | 0.4989 |
|  | cg02700891 | 3 | *TRH* | 5'UTR;1stExon | 1.37 (0.43, 2.31) | 0.0043 | 0.0456 | -0.12 (-0.96, 0.71) | 0.7725 |
|  | cg02861775 | 6 | *C6orf176* | Body | -2.83 (-4.63, -1.04) | 0.0020 | 0.0286 | 0.45 (-0.07, 0.97) | 0.0929 |
|  | cg03415695 | 22 | *LOC100271722* | Body | -3.37 (-5.66, -1.07) | 0.0040 | 0.0456 | 0.29 (-0.38, 0.96) | 0.4009 |
|  | cg04843085 | 11 | *C11orf45; KCNJ5* | Promoter; 5'UTR | 4.21 (1.13, 7.30) | 0.0075 | 0.0470 | 0.11 (-0.46, 0.69) | 0.6961 |
|  | cg05299847 | 21 | *CBS* | Body | 4.41 (2.27, 6.55) | 0.0001 | 0.0011 | 0.04 (-0.33, 0.42) | 0.8174 |
|  | cg05597624 | 1 | *RNF220* | 5'UTR | -2.23 (-3.82, -0.65) | 0.0058 | 0.0470 | -0.29 (-0.80, 0.22) | 0.2653 |
|  | cg06953601 | 6 | *TRAM2* | 5'UTR; 1stExon | -2.42 (-4.44, -0.40) | 0.0188 | 0.0470 | 0.56 (-0.14, 1.26) | 0.1175 |
|  | cg07957294 | 1 | *PRRX1* | Body | -3.49 (-5.45, -1.52) | 0.0005 | 0.0090 | 0.18 (-0.33, 0.68) | 0.4900 |
|  | cg08200446 | 19 | *ZNF44* | Intergenic | -2.17 (-3.85, -0.49) | 0.0115 | 0.0470 | 0.35 (-0.14, 0.84) | 0.1606 |
|  | cg08848958 | 16 | *BAIAP3* | Promoter | 1.73 (0.59, 2.86) | 0.0028 | 0.0369 | -0.33 (-0.76, 0.10) | 0.1297 |
|  | cg09707262 | 4 | *NEUROG2* | Promoter | -2.80 (-4.70, -0.90) | 0.0038 | 0.0456 | -0.3 (-1.16, 0.56) | 0.4881 |
|  | cg10626349 | 2 | *RPIA* | Intergenic | 3.37 (0.95, 5.79) | 0.0064 | 0.0470 | -0.08 (-0.84, 0.68) | 0.8370 |
|  | cg11479221 | 4 | *MTTP* | Intergenic | 4.00 (1.47, 6.52) | 0.0019 | 0.0286 | 0.22 (-0.43, 0.87) | 0.5149 |
|  | cg14185670 | 18 | *SEC11C* | Promoter | 3.81 (1.86, 5.77) | 0.0001 | 0.0026 | -0.93 (-2.23, 0.36) | 0.1589 |
|  | cg15836231 | 6 | *MAP3K7* | Intergenic | -1.85 (-3.18, -0.51) | 0.0067 | 0.0470 | 0.29 (-0.22, 0.81) | 0.2618 |
|  | cg18876084 | 6 | *CD2AP* | Intergenic | 3.15 (1.39, 4.91) | 0.0005 | 0.0086 | 0.51 (-0.21, 1.22) | 0.1658 |
|  | cg22697108 | 5 | *FGF18* | Intergenic | -2.27 (-4.04, -0.49) | 0.0125 | 0.0470 | -0.05 (-0.53, 0.43) | 0.8442 |
|  | cg23402920 | 2 | *EIPR1* | Intergenic | 3.06 (0.68, 5.44) | 0.0116 | 0.0470 | -0.37 (-0.85, 0.10) | 0.1241 |
|  | cg23987789 | 1 | *VAMP3* | Intergenic | 1.55 (0.46, 2.63) | 0.0052 | 0.0470 | 0.44 (0.09, 0.79) | 0.0148 |
| **FEV_1_/FVC** |  |  |  |  |  |  |  |  |  |
|  | cg01537494 | 1 | *MIR200B* | Promoter | 8.37 (3.92, 12.81) | 0.0002 | 0.0033 | -0.36 (-1.05, 0.34) | 0.3164 |
|  | cg03861217 | 2 | *KCNJ3* | Body | -5.00 (-8.17, -1.83) | 0.0020 | 0.0142 | -0.04 (-0.89, 0.82) | 0.9356 |
|  | cg07151560 | 1 | *WDR77* | Promoter | 7.34 (3.50, 11.18) | 0.0002 | 0.0028 | -1.13 (-2.25, 0.01) | 0.0490 |
|  | cg08116922 | 7 | *EGFR* | Intergenic | 2.73 (1.02, 4.44) | 0.0018 | 0.0142 | -0.10 (-0.73, 0.53) | 0.7646 |
|  | cg09367721 | 1 | *TIMM17A* | Body | 4.26 (1.96, 6.57) | 0.0003 | 0.0037 | -0.41 (-1.57, 0.74) | 0.4833 |
|  | cg09514174 | 1 | *OSCP1* | Promoter | 3.50 (1.48, 5.52) | 0.0007 | 0.0077 | -0.11 (-0.71, 0.48) | 0.7130 |
|  | cg13303654 | 10 | *ZEB1* | Body | 5.45 (2.32, 8.59) | 0.0006 | 0.0077 | 0.01 (-0.76, 0.75) | 0.9903 |
|  | cg19723734 | 7 | *MAD1L1* | 5'UTR | 2.36 (0.48, 4.24) | 0.0139 | 0.0417 | 0.37 (-0.33, 1.07) | 0.2985 |
|  | cg20805367 | 17 | *C17orf49* | Body | 1.55 (0.63, 2.48) | 0.0010 | 0.0096 | 0.04 (-0.26, 0.34) | 0.7861 |
|  | cg22152521 | 13 | *ZC3H13* | Body | 1.74 (0.44, 3.03) | 0.0088 | 0.0354 | -0.21 (-0.61, 0.2) | 0.3231 |
|  | cg22675660 | 17 | *RAP1GAP2* | Body | -4.59 (-7.03, -2.16) | 0.0002 | 0.0033 | 0.02 (-0.64, 0.68) | 0.9597 |
|  | cg23190164 | 12 | *LGR5* | Body | -2.64 (-4.31, -0.97) | 0.0020 | 0.0142 | -0.39 (-0.83, 0.06) | 0.0901 |
|  | cg24479027 | 17 | *ABR* | Promoter | -0.63 (-1.14, -0.11) | 0.0182 | 0.0417 | -1.94 (-4.11, 0.24) | 0.0807 |
|  | cg24595510 | 20 | *SCRT2* | 3'UTR | -2.44 (-4.07, -0.82) | 0.0032 | 0.0161 | -0.49 (-1.51, 0.53) | 0.3487 |
|  | cg25729401 | 17 | *CYTH1* | Intergenic | 2.02 (0.28, 3.75) | 0.0229 | 0.0417 | 0.42 (-0.48, 1.32) | 0.3614 |
|  | cg26757305 | 4 | *ATP10D* | Promoter | 3.58 (1.38, 5.78) | 0.0014 | 0.0127 | -0.46 (-1.20, 0.27) | 0.2172 |

Note to table S1: 1) *Genes located at intergenic location were not found in Illumina annotation file and were identified using online tool SNIPPER

3) Chr. no = Chromosome number; ORs = odds ratios; CIs = confidence intervals

**Table S2: DMRs (k=33) for lung function trajectory in relation to childhood DNA-M identified by DMRcate (FDR <0.05) method**

| **Lung function trajectory** | **Molecular location of the DMR**  **(chromosome: start – end)** | **Annotated Genes** | **^#^No. CpGs in the region** | **CpGs Name** | **Stouffer** |
| --- | --- | --- | --- | --- | --- |
| **Males** |  |  |  |  |  |
| **FVC** |  |  |  |  |  |
|  | chr6: 110011156- 110011999 | *FIG4, AK9* | 2 | cg01500097 | 1.15× 10^-177^ |
|  |  |  |  | cg02304879 |  |
|  | chr13: 112986154-112986635 | *LINC01044* | 4 | cg13988338 | 9.26× 10^-143^ |
|  |  |  |  | cg03486986 |  |
|  |  |  |  | cg17516572 |  |
|  |  |  |  | cg15627136 |  |
|  | chr11: 113660695-113660756 | *ATF4P4, RP11* | 2 | cg26945670 | 1.13× 10^-113^ |
|  |  |  |  | cg18517222 |  |
|  | chr1: 46859774-46859791 | *FAAH* | 2 | cg12671744 | 3.51× 10^-65^ |
| **FEV_1_** |  |  |  | cg16267850 |  |
|  | chr17: 28928406-28928453 | *SMURF2P1* | 2 | cg27394845 | 1.24× 10^-243^ |
|  |  |  |  | cg27034606 |  |
|  | chr2: 172974138-172974630 | *DLX2** | 2 | cg11557618 | 1.14× 10^-175^ |
|  |  |  |  | cg00076325 |  |
|  | chr16: 31147044-31147177 | *PRSS8* | 2 | cg08775835 | 1.67× 10^-55^ |
|  |  |  |  | cg18225844 |  |
|  | chr13: 37249426-37249450 | *SERTM1* | 2 | cg25217100 | 1.78× 10^-48^ |
|  |  |  |  | cg21121616 |  |
|  | chr6: 31148383-31148552 | *POU5F1* | 6 | cg09179646 | 3.27× 10^-42^ |
|  |  |  |  | cg26668675 |  |
|  |  |  |  | cg03078486 |  |
|  |  |  |  | cg22701603 |  |
|  |  |  |  | cg26818629 |  |
|  |  |  |  | cg09357589 |  |
|  | chr6: 74009041-74009455 | *KHDC1** | 2 | cg23238119 | 4.35× 10^-14^ |
|  |  |  |  | cg18080509 |  |
| **FEV_1_/FVC** |  |  |  |  |  |
|  | chr2: 20424423-20425395 | *SDC1* | 2 | cg05127937 | 1.03× 10^-280^ |
|  |  |  |  | cg18868357 |  |
|  | chr16: 84029457-84029584 | NECAB2* | 2 | cg22167353 | 4.14× 10^-235^ |
|  |  |  |  | cg27642027 |  |
|  | chr22: 39713008-39713062 | *SNORD83A* | 2 | cg01882930 | 1.07× 10^-140^ |
|  |  |  |  | cg22416002 |  |
|  | chr5: 177366867-177367013 | *RP11-1252I4.2* | 2 | cg02013239 | 1.38× 10^-100^ |
|  |  |  |  | cg07372500 |  |
|  | chr1: 47656137-47656140 | *PDZK1IP1* | 2 | cg07150145 | 3.78× 10^-100^ |
|  |  |  |  | cg02291556 |  |
|  | chr1: 223899845-223899998 | *CAPN2* | 2 | cg00339319 | 1.41× 10^-95^ |
|  |  |  |  | cg24150051 |  |
|  | chr7: 1003645-1004748 | *COX19** | 3 | cg08735705 | 3.69× 10^-79^ |
|  |  |  |  | cg07930192 |  |
|  |  |  |  | cg09363068 |  |
|  | chr6: 31846996-31847009 | *SLC44A4* | 2 | cg07363637 | 3.88× 10^-43^ |
|  |  |  |  | cg24707219 |  |
|  | chr1: 11761078-11761296 | DRAXIN* | 2 | cg16020346 | 3.60× 10^-29^ |
|  |  |  |  | cg21109485 |  |
|  | chr5: 23951555-23951696 | *C5orf17* | 2 | cg25381085 | 8.99× 10^-26^ |
|  |  |  |  | cg04663556 |  |
|  | chr17: 78865368-78865662 | *RPTOR** | 2 | cg00704970 | 1.76× 10^-15^ |
|  |  |  |  | cg24207068 |  |
|  | chr15: 99409360-99409506 | *IGF1R** | 2 | cg00098799 | 1.12× 10^-12^ |
|  |  |  |  | cg03380198 |  |
|  | chr2: 65593908-65594021 | *SPRED2* | 2 | cg09884146 | 1.99× 10^-7^ |
|  |  |  |  | cg26376241 |  |
| **Females** |  |  |  |  |  |
| **FVC** |  |  |  |  |  |
|  | chr1: 1022530 - 1022900 | *C1orf159* | 2 | cg24437834 | 1.51× 10^-139^ |
|  |  |  |  | cg19999567 |  |
|  | chr11: 68621969 - 68622177 | *CPT1A** | 2 | cg11245990 | 3.25× 10^-133^ |
|  |  |  |  | cg07511668 |  |
|  | chr11: 68658383 - 68658836 | *MRPL21** | 3 | cg21862992 | 4.99× 10^-7^ |
|  |  |  |  | cg06112835 |  |
|  |  |  |  | cg21963583 |  |
| **FEV_1_** |  |  |  |  |  |
|  | chr4: 113437801 - 113438462 | *NEUROG2, RP11* | 2 | cg07997294 | 6.18× 10^-158^ |
|  |  |  |  | cg09707262 |  |
|  | chr1: 7842369 - 7842406 | *PER3-003* | 2 | cg14511923 | 1.33× 10^-142^ |
|  |  |  |  | cg23987789 |  |
|  | chr8: 144416404 -144416485 | *TOP1MT* | 2 | cg23629150 | 1.13× 10^-115^ |
|  |  |  |  | cg12188860 |  |
| **FEV_1_/FVC** |  |  |  |  |  |
|  | chr16: 56715756 - 56716182 | *MT1X* | 2 | cg03551406 | 1.73× 10^-101^ |
|  |  |  |  | cg26802333 |  |
|  | chr17: 79905219 - 79905255 | *MYADML2* | 2 | cg01430970 | 1.69× 10^-123^ |
|  |  |  |  | cg25010115 |  |
|  | chr7: 73256414 - 73256416 | *WBSCR27* | 2 | cg02237119 | 1.91× 10^-17^ |
|  |  |  |  | cg03781731 |  |
|  | chr20: 3051954 - 3052221 | *OXT* | 2 | cg04731988 | 1.43× 10^-5^ |
|  |  |  |  | cg13285174 |  |

Note to table S2:

1. DMRcate annotates to UCSC RefGene from the Illumina annotation file
2. *Genes were not found in Illumina annotation file and were identified using online tool SNIPPER

**Figure S1: Circular plots of CpGs identified in DMRs (A) for males (B) for females.**

**(A)**


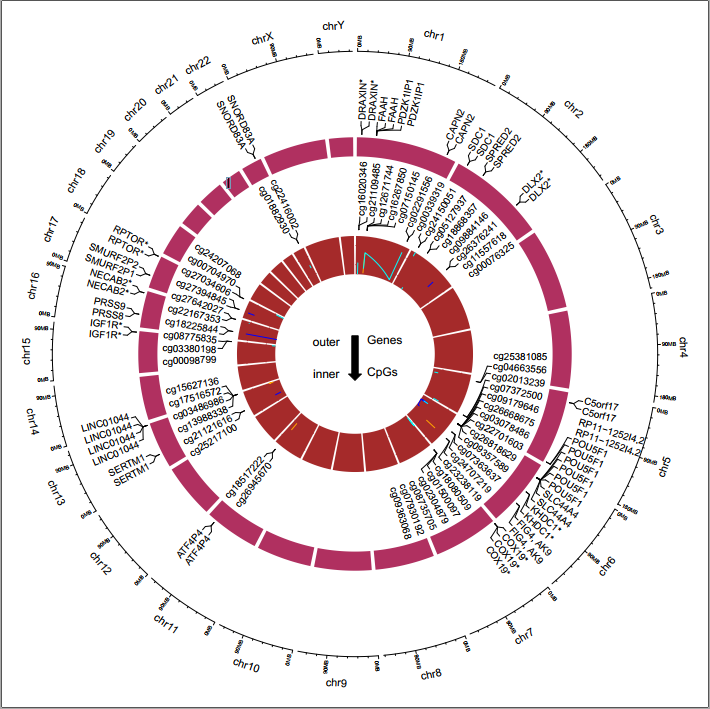


**(B)**


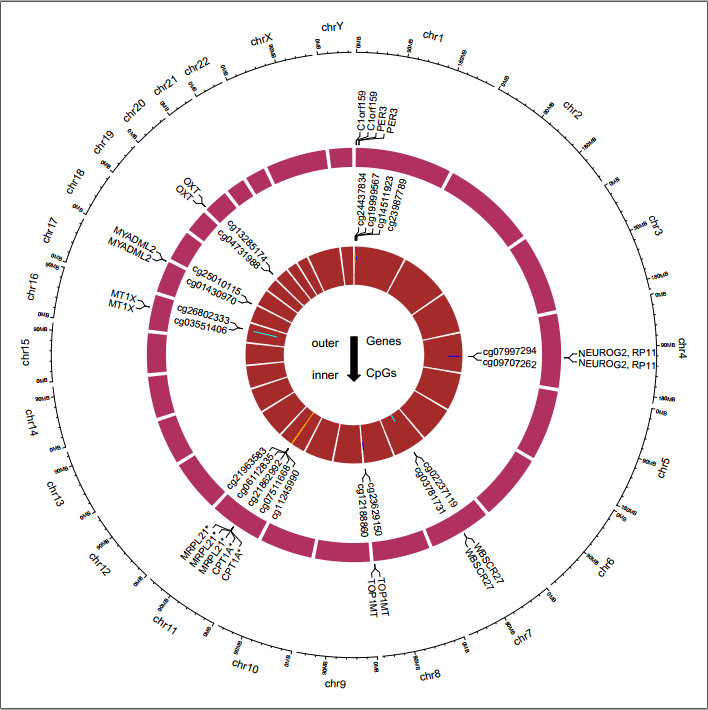


Note to figure S1: Chromosomes are shown in a clockwise direction from 1 to 22 in the outermost circle. The two innermost circles represent the gene and CpGs names for each lung function trajectory. Orange, blue, and aqua colors represent the P_FDR_ values of the DMRs identified for FVC, FEV_1_, and FEV_1_/FVC trajectories. *The corresponding CpGs are intergenic and the most adjacent genes’ name were identified from SNIPPER.
